# Diet and exercise orthogonally alter the gut microbiome and reveal independent associations with anxiety and cognition

**DOI:** 10.1186/1750-1326-9-36

**Published:** 2014-09-13

**Authors:** Silvia S Kang, Patricio R Jeraldo, Aishe Kurti, Margret E Berg Miller, Marc D Cook, Keith Whitlock, Nigel Goldenfeld, Jeffrey A Woods, Bryan A White, Nicholas Chia, John D Fryer

**Affiliations:** 1 Department of Neuroscience, Mayo Clinic, 4500 San Pablo Rd, Jacksonville, FL 32224, USA; 2 Department of Surgical Research, Mayo Clinic, 200 First St SW, Rochester, MN 55905, USA; 3 Departments of Kinesiology and Community Health, University of Illinois at Urbana-Champaign, Urbana, IL, USA; 4 Integrative Immunology and Behavior Group, University of Illinois at Urbana-Champaign, Urbana, IL, USA; 5 Institute for Genomic Biology, University of Illinois at Urbana-Champaign, Urbana, IL, USA; 6 Loomis Laboratory of Physics, University of Illinois at Urbana-Champaign, Urbana, IL, USA; 7 Center for Immunology and Immune Therapies, Mayo Clinic, 4500 San Pablo Rd, Jacksonville, FL 32224, USA; 8 Neurobiology of Disease Graduate Program, Mayo Clinic College of Medicine, 4500 San Pablo Rd., Jacksonville, FL 32224, USA

**Keywords:** Neuroscience, Gut-brain axis, Microbiome, Anxiety, Cognition, Exercise, Diet

## Abstract

**Background:**

The ingestion of a high-fat diet (HFD) and the resulting obese state can exert a multitude of stressors on the individual including anxiety and cognitive dysfunction. Though many studies have shown that exercise can alleviate the negative consequences of a HFD using metabolic readouts such as insulin and glucose, a paucity of well-controlled rodent studies have been published on HFD and exercise interactions with regard to behavioral outcomes. This is a critical issue since some individuals assume that HFD-induced behavioral problems such as anxiety and cognitive dysfunction can simply be exercised away. To investigate this, we analyzed mice fed a normal diet (ND), ND with exercise, HFD diet, or HFD with exercise.

**Results:**

We found that mice on a HFD had robust anxiety phenotypes but this was not rescued by exercise. Conversely, exercise increased cognitive abilities but this was not impacted by the HFD. Given the importance of the gut microbiome in shaping the host state, we used 16S rRNA hypervariable tag sequencing to profile our cohorts and found that HFD massively reshaped the gut microbial community in agreement with numerous published studies. However, exercise alone also caused massive shifts in the gut microbiome at nearly the same magnitude as diet but these changes were surprisingly orthogonal. Additionally, specific bacterial abundances were directly proportional to measures of anxiety or cognition.

**Conclusions:**

Thus, behavioral domains and the gut microbiome are both impacted by diet and exercise but in unrelated ways. These data have important implications for obesity research aimed at modifications of the gut microbiome and suggest that specific gut microbes could be used as a biomarker for anxiety or cognition or perhaps even targeted for therapy.

## Background

Obesity is a rising public health concern, especially in the United States where over a third of the population is categorized as obese [1]. It is a pervasive co-morbidity and is associated with a variety of diseases such as hypertension, stroke, coronary artery disease, diabetes, cancer, age-associated dementias, depression, and anxiety [2,3]. Obesity in children is associated with poorer academic achievement [4] and adult obesity is correlated with poorer performance on cognitive tasks [5-7]. Obese children and adults are more likely to have lower core self-evaluation and suffer from anxiety and depression [8]. Exercise is one proven approach to combat obesity in both humans and rodent models and likely operates via many potential mechanisms [9]. While many studies have demonstrated the positive effects of exercise on cognitive abilities or the anxiogenic effects of a high fat diet (HFD), virtually no studies have examined the intersection of these manipulations and whether exercise can normalize or prevent abnormalities of cognition or anxiety in a well-controlled animal model. This is of critical importance since some individuals assume that exercise can reverse all negative effects of a HFD including behavioral problems such as anxiety or impaired cognition.

We now know that obesity involves a number of factors and one of much recent interest has been the role of the gut microbiota in both weight gain and severe weight loss [10-13]. HFD is known to induce changes in the commensal gut bacterial community and seminal studies by the Gordon lab demonstrated that the gut microbiota are not merely reflective of the dietary intake, but they are actually key mediators of the metabolic state [11]. For example, fecal transfers from mice fed a HFD into naïve germ-free recipient mice led to increased adiposity in the recipients even when fed a normal diet (ND) [10]. However, very little is known about whether or how exercise affects the gut microbiome and whether alterations in the gut microbiome relate to changes in behavior. Interestingly, germ-free mice are less anxious than conventionally raised mice and re-colonization with a normal gut microbiota early in life is anxiogenic [14,15]. In this study we sought to determine the interaction of HFD and exercise on behavioral outcomes as well as the gut microbial community structure and define their relationships in adult mice. We hypothesized that HFD would cause behavioral problems and alter the gut microbiome but that the effects on both of these would be mitigated by exercise. Much to our surprise, we found that both HFD and exercise substantially altered behavior and the gut microbial community but exercise did not normalize or “rescue” the effects of a HFD and, in fact, the relationships were orthogonal.

## Results

### High fat diet causes anxiety that is not attenuated by exercise

**Figure 1:**
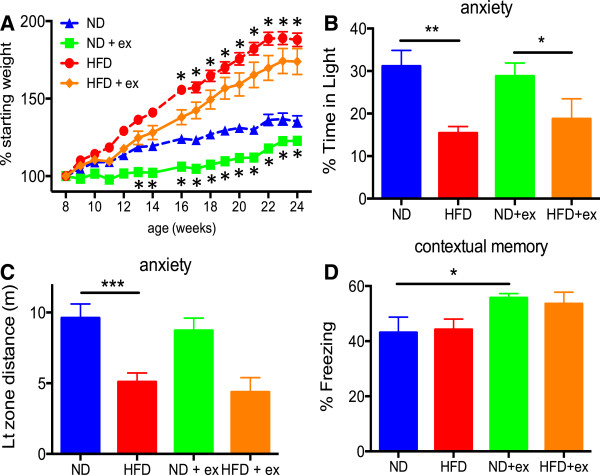
**Effects of diet and exercise on body weight, anxiety, and cognitive behavior. (A)** HFD-fed mice were significantly heavier starting at 4 weeks of treatment (ND vs HFD) while exercised mice weighed significantly less at 5 weeks of treatment for ND groups (ND vs ND + exercise, *p < 0.05) and at 8 weeks of treatment for HFD groups (HFD vs HFD + exercise, * p < 0.05). HFD-fed mice were more anxious as measured in the Light/Dark exploration assay for both percentage of time spent in the lit compartment **(B)** and distance traveled while in the lit compartment **(C)**. Exercised mice had enhanced learning and memory measured in the contextual fear conditioning assay **(D)**. Body weight analyzed by repeated measures ANOVA with post-hoc t-test and significance defined as p < 0.05. Behavioral data was analyzed by two-way ANOVA with post-hoc Fisher’s LSD with significance indicated as *p < 0.05, **p < 0.01, and ***p < 0.001. All data presented as mean +/- SEM from n=10/group.

To test the relationship between diet and exercise, we randomly assigned 8-week-old male C57BL/6 J mice to four groups: ND, ND + exercise, HFD, and HFD + exercise (n = 10/group). We chose a forced exercise paradigm so that we could precisely control the amount or “dose” of exercise. Importantly, to control for environmental enrichment and handling, non-exercised mice were placed along side their exercising counterparts in running wheels that rotated at a speed that just prevented them from sleeping (~1 m/min). As expected, HFD alone caused a significant increase in body weight compared to ND as early as 4 weeks after beginning treatment (Figure 1A). Exercised mice of both groups had significantly lower body weight than their sedentary counterparts beginning at 5 weeks of treatment for ND groups and 8 weeks of treatment for the HFD groups (Figure 1A). This amount of exercise was not able to completely counteract the effects of the HFD, but this was not surprising given the extremely high fat content of this diet (60% kcal from fat) and the limited amount of exercise from these one hour sessions.

We performed behavioral testing of these groups to determine the impact of HFD or exercise on anxiety and cognition. Open field analysis did not demonstrate any significant differences in total activity or rearing among the groups (Additional file 1: Figure S1). We also found no differences between the groups in sociability as measured in the three-chamber social interaction test (Additional file 1: Figure S1). Using the light/dark exploration apparatus as a measure of anxiety, we found that mice fed a HFD were significantly more anxious as measured by percentage of time spent in the light compartment and in distance traveled while occupying the light compartment (Figure 1B and C). However, exercise was not able to counteract these anxiogenic effects of the HFD (Figure 1B and C).

### Exercise improves memory but is not impacted by high fat diet

We next measured learning and memory in the contextual fear conditioning paradigm where mice are placed in a chamber and a loud tone is paired with a negative stimulus (foot shock). Mice that remember the environment will spend more time freezing when placed again in the same apparatus (contextual memory). Exercised mice on a normal diet had significantly increased contextual memory compared to their sedentary counterparts (Figure 1D). Exercised mice on a HFD diet had a trend toward increased contextual memory compared to their sedentary HFD counterparts, but this effect did not quite achieve statistical significance (Figure 1D). Similar results were obtained with the cued portion of the task (Additional file 1: Figure S1). However, HFD alone did not substantially alter either contextual or cued memory nor did it significantly reduce the positive effects of exercise.

### Effect of diet and exercise on the gut microbiome

**Figure 2:**
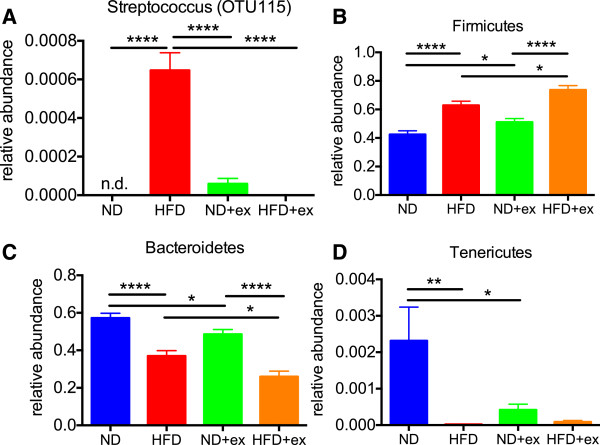
**Changes in the relative abundances of gut bacteria by HFD or exercise. (A)** HFD caused a bloom of OTU115 from the genus Streptococcus that returned to nearly undetectable levels in HFD + exercise mice. Bacterial of phyla Firmicutes **(B)**, Bacteroidetes **(C)**, and Tenericutes **(D)** of the gut microbiome were significantly altered by both HFD and exercise. Data was analyzed by two-way ANOVA with post-hoc Holm-Sidak t-tests with significance indicated as *p < 0.05, **p < 0.01, ***p < 0.001, and ****p < 0.0001. All data presented as mean +/- SEM from n=10/group.

We also assessed whether exercise alone or in the context of a HFD could alter the gut microbial community. We purified DNA from fecal samples from each of the mice and profiled their gut microbiome using next generation barcoded sequencing of amplicons from the variable regions V3-V5 of the bacterial 16S ribosomal DNA (a “fingerprint” of different bacterial taxa). A total of 35,197,991 reads were obtained from 40 samples (averaging 879,950 ± 249,310 reads; minimum 431,900 reads; maximum 1,416,925 reads) using a 300-cycle kit on a single Illumina MiSeq sequencing run. Paired reads were then analyzed using an extension of the TORNADO pipeline for taxa, operational taxonomic unit (OTU), and phylogeny [16]. QIIME [17,18] was used to calculate beta-diversity, which was then visualized using R with the vegan package for ordination plots.Our initial hypothesis was that exercise would rescue many of the shifts in the gut microbiome caused by a HFD. While we found a few examples where exercise modulated HFD-induced changes in the microbiome, including a remarkable “bloom” of a Streptococcus genus (OTU115, family Streptococcaceae, order Lactobacillales) that was returned to undetectable levels with exercise (Figure 2A), the majority of microbiome alterations exerted by diet and exercise followed an unexpected pattern. Not only did exercise alone cause large shifts in the two most abundant phyla, Firmicutes and Bacteroidetes, but the directionality and magnitude of the effect was similar to the changes caused by HFD (Figure 2B,C). Both diet and exercise also independently decreased the relative abundance of Tenericutes (Figure 2D).

**Figure 3:**
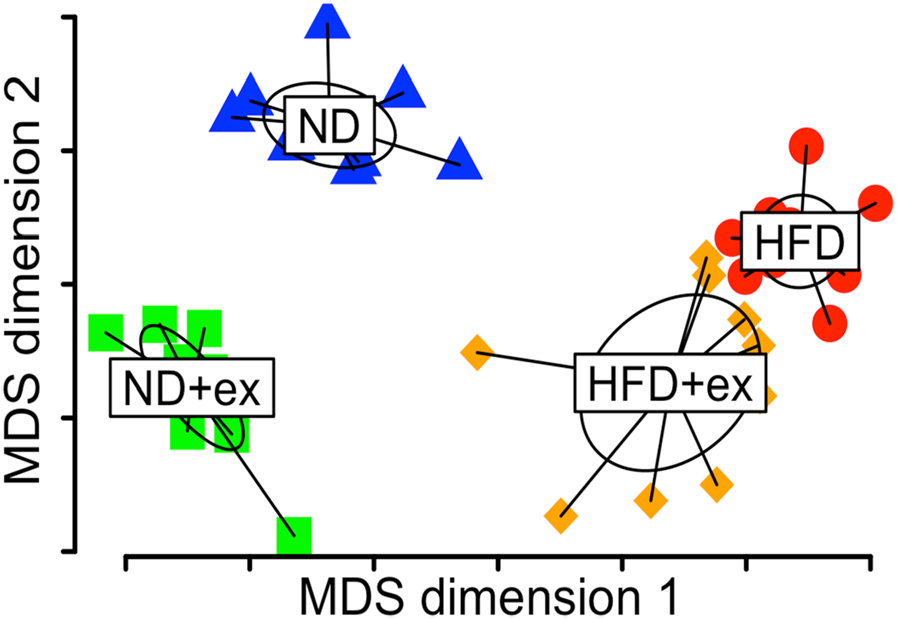
**Multidimensional analysis of diet and exercise reveals orthogonal changes in the gut microbiome.** This analysis in multidimensional space demonstrates clear segregation of each of the four groups of mice with no overlap between groups.

**Figure 4:**
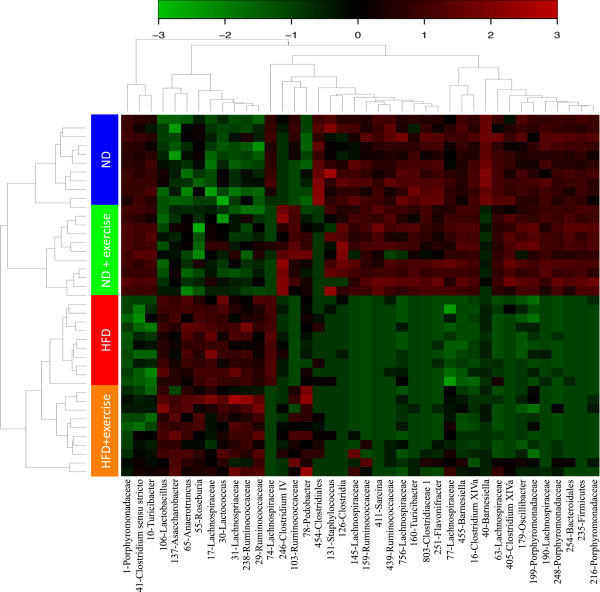
Heat map of global analysis of diet and exercise reveals multiple distinct signatures with clear dietary effects and secondary exercise effects.

While the changes in phyla induced by HFD or exercise appear similar, this broad grouping of bacteria does not provide the resolution for determining the effects of diet or exercise on the community structure. In order to refine the analysis, we analyzed these data at the OTU level with each OTU representing bacteria grouped at 97% identical sequences from the 16S sequencing (see Methods). Metric Dimensional Analysis (MDA) is one such way to visualize similarities or dissimilarities in large data sets while preserving their distance relationships. MDA revealed that a high percentage of variation is explained by just the first and second principal components with each of the four groups of mice separating rather cleanly into four distinct clusters (Figure 3). This indicates that most diet- or exercise-induced shifts in microbial populations are completely unrelated (i.e. orthogonal). More detailed examination of what drives this separation in ordination space revealed multiple strong signatures separating ND from HFD groups and secondarily separating sedentary from exercised groups (Figure 4 with OTU probabilities shown in Additional file 2: Table S2). Additionally, we analyzed different levels of taxonomy and found significant main effects of diet on the abundance of many different families and an equal number of main effects of exercise (Table 1). Several main effects of diet or exercise were also seen at higher levels of taxonomy such as order, class, and phylum (Additional file 3: Table S1).

**Table 1:** Significant effects of diet and exercise at the level of family

	**Main effects 2 way ANOVA**			**Post-hoc testing**			
				**ND vs**	**ND + ex vs**	**ND vs**	**HFD vs**
**Family**	**High fat diet**	**Exercise**	**Interaction**	**HFD**	**HFD + ex**	**ND + ex**	**HFD + ex**
Porphyromonadaceae	↓ ****	↓ ***		****	****	*	*
Streptococcaceae	↑ ****	↓ **	*	****	**		**
Lachnospiraceae	↑ ****	↑ *		****	****		
Erysipelotrichaceae	↓ ****			**	***		
Clostridiaceae_1	↑ ****			**	****	*	
Ruminococcaceae	↑ ****				***		
Peptostreptococcaceae	↑ ***	↑ ***	**		***		***
Eubacteriaceae	↑ ***		*		**		
Peptococcaceae_1	↓ **	↓ **	**	**		**	
Anaeroplasmataceae	↓ **		*	**		*	
Staphylococcaceae	↓ **						
Cryomorphaceae	↑ *	↑ **	*		**	**	
Phyllobacteriaceae	↑ *	↑ *			*		*
Alcaligenaceae	↑ *	↑ *					
Pseudomonadaceae	↑ *	↑ *			*		*
Bacteroidaceae	↑ *			*			
Veillonellaceae	↑ *						
Enterobacteriaceae	↑ *						
Lactobacillaceae	↑ *						
Gracilibacteraceae	↑ *						
Peptococcaceae_2		↓ ***				*	
Rhizobiaceae		↑ **					
Incertae_Sedis_IV		↑ **				*	
Microbacteriaceae		↑ *					
Nocardiaceae		↑ *					
Coriobacteriaceae		↑ *				*	
Flavobacteriaceae		↑ *					*
Sphingobacteriaceae		↑ *			*		*
Bradyrhizobiaceae		↑ *					
Caulobacteraceae		↑ *					*
Burkholderiaceae		↑ *					*
Comamonadaceae		↑ *					

*P<0.05, **P<0.01, ***P<0.001, ****P<0.0001.

### Associations between specific taxa with anxiety and cognition

**Figure 5:**
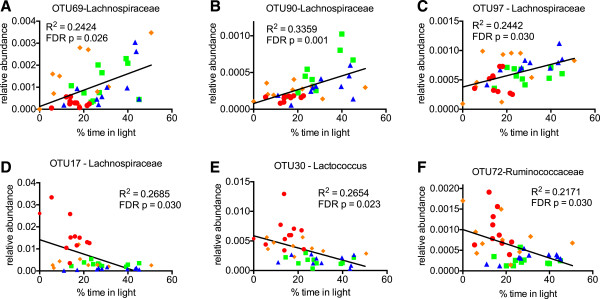
**Several bacteria significantly associate with anxiety measures.** Relative abundances of OTU69, 90, and 97 positively correlated with % time in light in the Light/Dark test **(A-C)** while OTU17, 30, and 72 negatively correlated with % time in light **(D-F)**. Most likely family or genus shown on graph. Color scheme of data points follows the format as in Figure 1 (ND = blue, ND + exercise = green, HFD = red, and HFD + exercise in orange). Linear regression analysis for individual mice was performed with R^2^ values indicating goodness of fit and p values (indicated on graph) for slope calculated by F test with Benjamini-Hochberg correction for false discovery rate.

**Figure 6:**
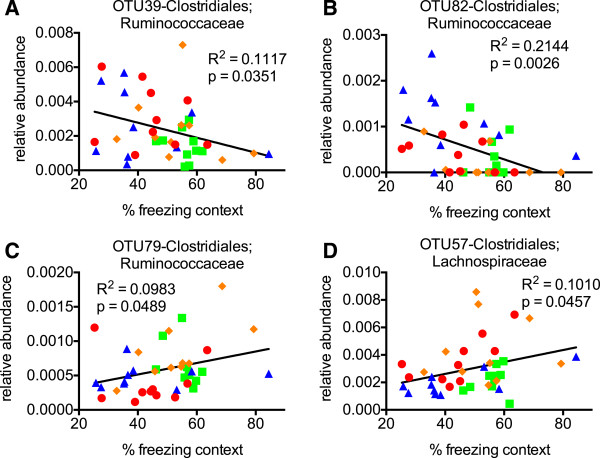
**Several bacteria specifically associate with cognitive measures. (A-D)** The relative abundance of OTU’s 39 and 82 negatively correlated with % time freezing in the contextual fear conditioning test **(A, B)** while OTU79 and 57 positively correlated with % time freezing **(C, D)**. Most likely family or genus shown on graph. Color scheme of data points follows the format as in Figure 1 (ND = blue, ND + exercise = green, HFD = red, and HFD + exercise in orange). Linear regression analysis for individual mice was performed with R^2^ values indicating goodness of fit and uncorrected p values (indicated on graph) for slope calculated by F test.

We next wanted to test whether specific taxa would correlate with behavioral measures. We analyzed the top 100 OTU’s and found that the relative abundance of OTU69, OTU90, and OTU97, all from the family Lachnospiraceae, positively correlated with % time in light, that is higher levels correlated with less anxiety (Figure 5). Other specific bacteria such as OTU17 (family Lachnospiraceae), OTU30 (family Streptococcaceae, genus Lactococcus), OTU72 (family Ruminococcaceae), and OTU89 (family Ruminococcaceae, genus Butyricicoccus) negatively correlated with% time in light, that is lower levels correlated with less anxiety (Figure 5). In fact, anxiety relationships were found at all levels of taxonomy including family, order, class, and even phylum (Additional file 4: Figure S2), although with these sample sizes they did not reach statistical significance when corrected for false discovery rate. We also found weaker associations with cognitive performance between bacteria from the order Clostridiales such as OTU39, 82, 79, and 57 (Figure 6), although these also did not quite reach significance when corrected for false discovery rate. Nevertheless, it is interesting that they all fell within a single order (Clostridiales). In agreement with numerous other studies, several highly significant relationships with body weight were seen at all levels of taxonomy including the phyla Bacteroidetes, Firmicutes, and Proteobacteria (Additional file 5: Table S3).

## Discussion

Very few studies have examined how exercise alone impacts the gut microbiome. Voluntary exercise in rats caused global changes in the gut microbiome but the technique used in those studies (PCR banding patterns) is only useful in determining whether differences exist and cannot be used to ascertain which bacteria are altered [19,20]. One study found that voluntary exercise in mice caused large alterations in the gut microbiome and with clear separations in ordination space [21], but the techniques used (PhyloChip arrays) make it difficult or impossible to compare to our data given the differences in accurately assigning bacteria to different taxa. Interestingly, one study that employed deep sequencing techniques failed to find significant changes in the gut microbiome [22], but they used voluntary wheel running and their first sampling of the microbiome was after 1 year of exercise compared to our changes found at 16 weeks. A recent study of similar design and duration to ours also found that diet and exercise exerted orthogonal effects on the gut microbiota [23]. However, they reported that exercise increased Bacteroidetes and decreased Firmicutes (opposite of our results) perhaps due to their voluntary wheel running paradigm [23].

Due to the nature of studying the interaction of exercise and diet, which requires subjects to be fed a high fat diet as one of the conditions, it is unlikely that similar studies will ever be conducted in humans. Therefore, it is important to understand how these factors interact in a well-controlled animal model system in order to determine how these parameters may impact human health. Although numerous studies have shown the benefits of exercise on cognitive function [7,24,25] as we have demonstrated here, few studies have actually examined the interaction between HFD and exercise, specifically in regard to behavioral outcomes. Rat studies have suggested that exercise can partially rescue the cognitive decline associated with HFD; however, an important parameter of behavior, anxiety, was not measured in these studies [26,27]. Our current study clearly indicates that exercise alone is unable to counteract all of the effects of a HFD. This is a critical finding given that some humans likely operate under the assumption that exercise can completely reverse the negative impact of HFD and change how we view weight loss that is conducted through exercise alone.

Somewhat to our surprise, our data suggest that HFD and exercise independently impact the different behavioral domains of anxiety and cognition, much like the orthogonal effects on the gut microbiome. Though the cognition associations with specific OTUs were weaker compared to anxiety, we nonetheless found significant relationships that all fell within Clostridiales, a diverse taxonomic order within the phyla Firmicutes that has not been previously associated with cognition. Clearly further studies with larger samples sizes are required to confirm these associations.

Numerous studies have described how the gut microbiome is altered by a HFD and obesity including changes in Firmicutes and Bacteroidetes [28-31]. Though only a very minor phylum in terms of representation, we also found that HFD reduced the relative abundance of Tenericutes, driven almost entirely by a single OTU (#67) from the genus Anaeroplasma (>99% probability as shown in Additional file 2: Table S2) that has not been previously implicated in dietary manipulations. HFD induced large changes at all levels of taxonomy, including in the two most abundant classes/orders (Bacteroidia/Bacteroidales and Clostridia/Clostridiales). We also found HFD caused many significant changes in taxa of more minor abundance. For example, highly significant alterations were seen for several orders (e.g. Lactobacillales, Erysipelotrichales, and Anaeroplasmatales) and classes (e.g. Erysipelotrichia, Mollicutes, and Bacilli). Subsequent studies will determine which of these are the most important players, but probiotic studies support the idea that even taxa of minor abundance can still elicit large effects on the host. Current methods of bacterial profiling as we have used here do not provide sufficient coverage of the entire 16S region to reliably identify changes at the species level, but such studies would allow for more direct translational potential of findings (e.g. pre- or probiotics).

Many studies have documented the negative consequences of dietary fat intake and obesity on behavior and brain function in rodents [32-36]. Diet-induced obesity has been shown to promote depressive-like behavior due to altered brain reward circuitry in mice [32]. Interestingly, caloric restriction has been shown to reduce anxiety and depression related behaviors in mice [37]. One study demonstrated HFD-induced anxiety behavior and changes in the gut microbiome at the level of phylum, but the techniques used did not allow for high resolution analysis [38]. In addition to low anxiety phenotypes in germ-free mice [14,15], some studies have shown that modulation of the gut microbiota either by diet or probiotics can impact anxiety [39,40]. The majority of anxiety associations from our study were a subset of the same taxa that associated with body weight. Interestingly, alterations of Ruminococcaceae and Lachnospiraceae were found in mice exposed to a grid floor stress paradigm [41]. We found a few associations with anxiety that were independent of body weight alterations, including OTU37 (Ruminococcaceae; Clostridium_IV), OTU40 (Barnesiella), and OTU48 (Eubacterium), and these as well as OTU30 (Lactococcus) have no reported associations with anxiety. It is important to point out that we cannot establish causality from these studies. Indeed, others have found that manipulations that cause anxiety in rodents can also result in changes to the gut microbial community [42,43]. These “top down” vs “bottom up” dynamics between the CNS and the gut microbiota require further study to fully delineate the causality, but it is highly likely that both mechanisms are at play with some feedback at both levels (i.e. a true system). Likewise, further studies on high fat diet and exercise are required to determine whether these specific bacteria are biomarkers of anxiety or actually drivers of the anxiety phenotypes or both.

## Conclusions

In this study we found that a HFD was able to cause significant anxiety with no rescue by exercise while exercise, but not HFD, was able to enhance cognition. We also found that exercise alone robustly altered the gut microbial community and did not rescue the changes induced by a HFD but, in fact, the changes caused by exercise were completely orthogonal to those induced by a HFD. Additionally, we found numerous, independent associations of specific OTUs and taxa with body weight, anxiety, and cognition that will need to be empirically tested to determine their importance. These data have important public health implications not only in terms of obesity but also determining how the gut microbial community relates to behavioral domains and how it may be used as a biomarker or reshaped to effect changes in anxiety and cognition.

## Methods

### Mice and treatments

Adult male C57BL/6 J mice were purchased from Jackson Laboratories (Bar Harbor, MN) and were randomly assigned to n = 10/group . Beginning at 8 weeks of age, mice were randomized to one of four groups: ND, ND + exercise, HFD, and HFD + exercise. Diet consisted of normal diet (10% of calories from fat; Research Diets, New Brunswick, NJ #D12450B) or high fat diet (60% of calories from fat; Research Diets #D12492). After an initial 2 week training period where the speed was gradually increased from 3 to 7 m/min and duration from 6 to 60 minutes per day, exercised groups were placed in running wheels (Lafayette Instruments, Lafayette, IN) for one hour at 7 m/min every morning for 5 days/week for the remainder of the study with the exception of the behavioral testing days. Control “sedentary” groups were placed in adjacent running wheels that rotated at a speed that just prevented them from sleeping (~1 m/min) to control for environmental enrichment and handling. All procedures were in accordance with guidelines from the Institutional Animal Care and Use Committee under an approved protocol.

### Behavioral analysis

For all behavioral tests, mice were acclimated in the room for an hour prior to the onset of testing. Data was recorded and monitored using overhead cameras and tracked with Anymaze software (Stoelting Co., Wood Dale, IL). Three days following the last exercise session, mice were tested on consecutive days in the open field assay, light/dark box, three-chamber social apparatus, and contextual fear conditioning (over two days) always in the first half of the light phase.

### Open field assay (OFA)

Mice were placed in a 40 × 40 × 30 cm (W × L × H) opaque Perspex box and activity was tracked during the entire 15 minute observation period.

### Light/Dark exploration (LDE) assay

Mice were placed in a square 40 × 40 × 30 cm Perspex box equally divided into two compartments with a small open door joining the light and dark compartments. The dark compartment was completely covered and mice were tested by placing them at the far end of the lit chamber facing away from the dark chamber and their activity was tracked for 10 minutes.

### Three chamber social interaction (3SI)

Social behavior was tested in a three-chamber Perspex 40 × 40 cm box consisting of two 17x40 cm regions separated by 2 dividers forming a smaller 5 × 40 cm center region. Mice were able to move freely through a small 8 × 5cm opening that was aligned in both dividers. Each of the larger chambers contained an inverted wire mesh cylinder in opposing corners. Mice were initially acclimated to the box and empty cylinders for 4 min and then placed in temporary holding cages. A male probe mouse of the same strain was placed in one of the inverted mesh cylinders and allowed to acclimate for 3 minutes prior to placing the test mouse back in the box. The open mesh enabled visual, olfactory, and auditory interactions between probe and test mice. Test mice were subsequently monitored for 10 min in the presence of the probe mouse and interaction score was calculated with the following formula: (Time Mouse cup – Time empty cup)/ (Time Mouse cup + Time empty cup).

### Contextual fear conditioning assay (CFC)

This test was conducted in a sound attenuated chamber with a grid floor capable of delivering an electric shock and freezing was measured with an overhead camera and FreezeFrame software (Actimetrics, Wilmette, IL). Mice were initially placed into the chamber and undisturbed for 2 minutes, during which time baseline freezing behavior was recorded. An 80-dB white noise served as the conditioned stimulus (CS) and was presented for 30 sec. During the final 2 sec of this noise, mice received a mild foot shock (0.5 mA), which served as the unconditioned stimulus (US). After 1 minute, another CS-US pair was presented. The mouse was removed 30 sec after the second CS-US pair and returned to its home cage. Twenty-four hours later, each mouse was returned to the test chamber and freezing behavior was recorded for 5 minutes (context test). Mice were returned to their home cage and placed in a different room than previously tested in reduced lighting conditions for a period of no less than one hour. For the auditory CS test, environmental and contextual cues were changed by: wiping testing boxes with 30% isopropyl alcohol instead of 30% ethanol; replacing white house lights with red house lights; placing a colored plastic triangular insert in the chamber to alter its shape and spatial cues; covering the wire grid floor with opaque plastic; and altering the smell in the chamber with vanilla extract. The animals were placed in the apparatus for 3 min and then the auditory CS was presented and freezing was recorded for another 3 min (cued test). Baseline freezing behavior obtained during training was subtracted from the context or cued tests to control for variability in each animal.

### Fecal DNA isolation and 16S amplicon preparation and sequencing

Fecal samples left behind in the open field apparatus were collected and immediately frozen on dry ice until processing. Fecal DNA was isolated using a PowerSoil kit (MoBio, Carlsbad, CA) according to directions.

Amplification targeted V3 and V5 regions of the bacterial 16S subunit [44] using primers 357 F (AATGATACGGCGACCACCGAGATCTACACTATGGTAATTGTCCTACGGGAGGCAGCAG) and 926R (CAAGCAGAAGACGGCATACGAGAT-NNNNNNNNNNNN-AGTCAGTCAGCCCCGTCAATTCMTTTRAGT) with barcodes 1–40 from Caporaso et al [45]. PCR was run through 34 cycles of 98°C for 15 seconds, 70°C for 20 seconds, 72°C for 15 seconds with Kapa Hotstart Hi-Fi (Kapa Biosystems, Boston, MA). Electrophoresis of a small sample was used to verify amplicon specificity and purification was carried out using magnetic beads. DNA for each amplicaon was then diluted to 10nM and pooled for sequencing on a MiSeq (Illumina, San Diego, CA) using a 300 cycle kit and custom read1 (TATGGTAATTGTCCTACGGGAGGCAGCAG), read2 (AGTCAGTCAGCCCCGTCAATTCMTTTRAGT), and index (ACTYAAAKGAATTGACGGGGCTGACTGACT) sequencing primers.

### Pipeline for processing of 16S data

Pre-processed sequence files are then subject to quality filtering using Trimmomatic [46] version 0.22, with a hard cutoff of PHRED score Q3 for 5′ and 3′ ends of the reads (parameters LEADING:3 and TRAILING:3), trimming of the 3′ end with a moving average score of Q15, with a window size of 4 bases (parameter SLIDINGWINDOW:4:15), and removing any remaining reads shorter than 75\% of the original read length (parameter MINLEN:112 for reads of 150 bp long). Finally reads with any ambiguous base calls or with homopolymers longer than 10 bases longs are discarded using mothur [47]. Only the read pairs that survived the quality filter were processed further. Any surviving reads that were unpaired (that is, they lost their matching pair due to low quality) were discarded. Surviving read pairs were then grouped into two files, one each for “read 1” and “read 2” sequences. Reads were also dereplicated, consolidating identical reads to avoid redundant processing. Each of the two read libraries was then checked for chimeras using UCHIME in de novo mode.

### Taxonomy assignment

To prepare the reads for this step, we took reads from the previous step just before the stitching procedure, remove the gaps and then stitched them with a pad sequence of “N” bases. The specific scripts used for these file manipulations are publically available as part of the IM-TORNADO 16S rRNA analysis pipeline (http://sourceforge.net/projects/imtornado/). Since by default most Bayesian classifiers use 8-mers to perform the classification, we used “NNNNNNNN” as the padding. The stitched reads were then classified using the Greengenes taxonomy (Greengenes99 database version 12.10) as the reference [48] using the following mothur “classify.seqs” command with “iter = 1000”.

### Clustering, representative sequences, and chimera removal

Paired-end reads were concatenated directly with no padding and dereplicated. OTU representatives were selected and used to generate a reference set for clustering using USEARCH command “usearch7.0.1090_i86linux32” [49]. Clustering width was set at 97% (−otu_radius_pct 3.0).

### Ordination plot

Distance between communities was measured using unweighted Unifrac as implemented in QIIME version 1.8 [16]. Ordination plot was generated using multidimensional scaling analysis as implemented in R and displayed using the vegan package for ecological analyses (http://CRAN.R-project.org/package=vegan).

### Heatmap

METAGENassist [50] heatmap was produced using the 40 most significant OTU features. Data was log normalized in METAGENasssist. Clustering of the groups was carried out using complete linkage clustering and the Spearman distance metric.

### Correlation analysis

Relative abundances of bacteria from different levels of taxonomy were analyzed by linear regression with R^2^ values indicating goodness of fit and p values for slope calculated by F test with Benjamini-Hochberg correction for false discovery rate (FDR) where indicated.

## Competing interests

The authors declare that they have no competing interests.

## Authors’ contributions

SSK, NC, and JDF designed and performed this study, analyzed data, and wrote the manuscript. PRJ, MEBM, MDC, KW, NG, JAW, and BAW assisted in purification, sequencing, and analysis of fecal DNA. AK assisted in behavioral analysis. All authors discussed the results and provided comments on the manuscript. All authors read and approved the final manuscript.

## Supplementary Material

Additional file 1: Figure S1 Impact of diet and exercise on other mouse behaviors. Neither HFD nor exercise altered total locomotor activity (A) or rearing (B) in the open field assay or in sociability (C) in the three chamber social test. As with the contextual memory, exercised mice had a trend toward increased cued memory (D, p = 0.051) in the cued portion of the contextual fear conditioning assay. Click here for file

Additional file 2: Table S2 OTU and probabilities of correct taxonomic classification (in parentheses). Click here for file

Additional file 3: Table S1 Significant effects of diet and exercise at different levels of taxonomy. *P<0.05, **P<0.01, ***P<0.001, ****P<0.0001. Click here for file

Additional file 4: Figure S2 Anxiety levels were associated with several bacteria at the level of phyla, class, order, and family. Color scheme of data points follows the format as in Figure 1 (ND = blue, ND + exercise = green, HFD = red, and HFD + exercise in orange). Linear regression analysis for individual mice was performed with R2 values indicating goodness of fit and uncorrected p values for slope calculated by F test. Click here for file

Additional file 5: Table S3 Associations of bacterial abundances with body weight. *P<0.05, **P<0.01, ***P<0.001, ****P<0.0001. Click here for file
