# Supplementary material for: Diet and exercise orthogonally alter the gut microbiome and reveal independent associations with anxiety and cognition
Source: Mol Neurodegener. 2014 Sep 13;9:36. doi: 10.1186/1750-1326-9-36 (PMC4168696; doi:10.1186/1750-1326-9-36)
Supplement: Additional file 1: Figure S1 — Impact of diet and exercise on other mouse behaviors. Neither HFD nor exercise altered total locomotor activity (A) or rearing (B) in the open field assay or in sociability (C) in the three chamber social test. As with the contextual memory, exercised mice had a trend toward increased cued memory (D, p = 0.051) in the cued portion of the contextual fear conditioning assay. [file 1750-1326-9-36-S1.pdf]

Supplemental Figure 1

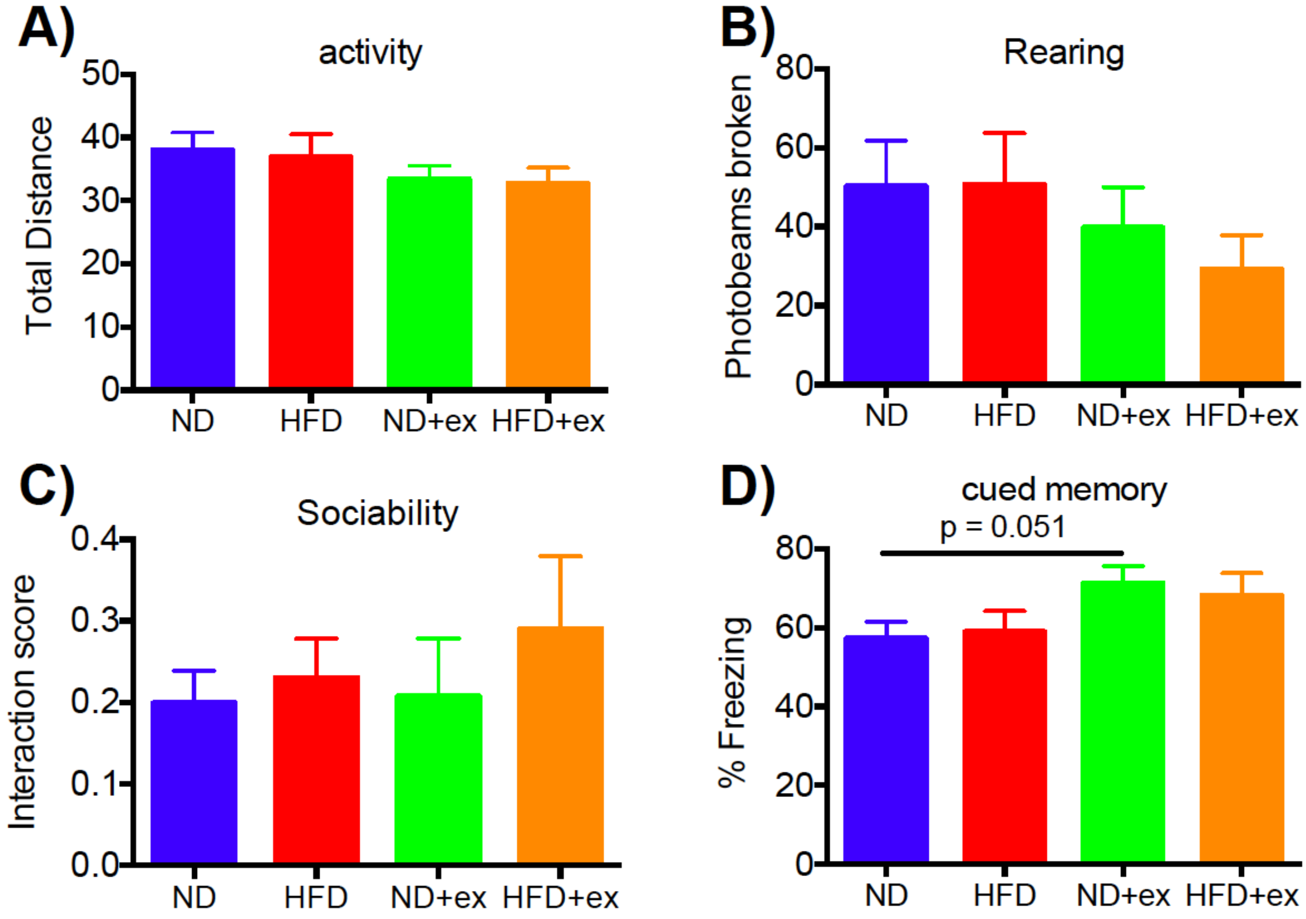

Figure S1. Impact of diet and exercise on other mouse behaviors. Neither HFD nor exercise altered total locomotor activity (A) or rearing (B) in the open field assay or in sociability (C) in the three chamber social test. As with the contextual memory, exercised mice had a trend toward increased cued memory (D, $p = 0.051$) in the cued portion of the contextual fear conditioning assay.
