# Supplementary material for: Diet and exercise orthogonally alter the gut microbiome and reveal independent associations with anxiety and cognition
Source: Mol Neurodegener. 2014 Sep 13;9:36. doi: 10.1186/1750-1326-9-36 (PMC4168696; doi:10.1186/1750-1326-9-36)
Supplement: Additional file 2: Table S2 — OTU and probabilities of correct taxonomic classification (in parentheses). [file 1750-1326-9-36-S2.pdf]

Supplemental Table S2. OTU and probabilities of correct taxonomic classification (in parentheses)

OTU	Taxonomy probabilities
1	Bacteria(100);"Bacteroidetes"(99.9);"Bacteroidia"(98.9);"Bacteroidales"(98.9);"Porphyromonadaceae"(94);Barnesiella(51.7);
2	Bacteria(100);"Bacteroidetes"(100);"Bacteroidia"(98);"Bacteroidales"(98);"Porphyromonadaceae"(96.6);Barnesiella(87.6);
3	Bacteria(100);"Bacteroidetes"(100);"Bacteroidia"(99.6);"Bacteroidales"(99.6);"Porphyromonadaceae"(97.7);Barnesiella(74.7);
4	Bacteria(100);Firmicutes(99);Clostridia(98.9);Clostridiales(98.9);Lachnospiraceae(90.5);Syntrophococcus(22.5);
5	Bacteria(100);Firmicutes(99.6);Clostridia(99.6);Clostridiales(99.6);Lachnospiraceae(95.5);Clostridium_XlVa(32.9);
6	Bacteria(100);"Bacteroidetes"(100);"Bacteroidia"(99.2);"Bacteroidales"(99.2);"Porphyromonadaceae"(96.9);Barnesiella(52.9);
7	Bacteria(100);Firmicutes(100);Clostridia(100);Clostridiales(100);Ruminococcaceae(100);Oscillibacter(94.4);
8	Bacteria(100);Firmicutes(100);Clostridia(100);Clostridiales(100);Ruminococcaceae(100);Oscillibacter(90.5);
9	Bacteria(99.9);Firmicutes(99.9);Clostridia(99.9);Clostridiales(99.9);Lachnospiraceae(98.4);Marvinbryantia(26.6);
10	Bacteria(100);Firmicutes(100);Erysipelotrichia(100);Erysipelotrichales(100);Erysipelotrichaceae(100);Turicibacter(100);
11	Bacteria(100);Firmicutes(100);Clostridia(100);Clostridiales(100);Ruminococcaceae(100);Anaerotruncus(43.9);
12	Bacteria(100);Firmicutes(100);Clostridia(100);Clostridiales(100);Peptostreptococcaceae(100);Clostridium_XI(100);
13	Bacteria(100);Firmicutes(99.8);Clostridia(99.7);Clostridiales(99.7);Eubacteriaceae(59.5);Eubacterium(59.5);
14	Bacteria(100);Firmicutes(100);Clostridia(100);Clostridiales(100);Ruminococcaceae(100);Pseudoflavonifractor(87.6);
15	Bacteria(100);"Bacteroidetes"(100);"Bacteroidia"(98.5);"Bacteroidales"(98.5);"Porphyromonadaceae"(97.9);Barnesiella(68.2);
16	Bacteria(100);Firmicutes(100);Clostridia(100);Clostridiales(100);Lachnospiraceae(100);Clostridium_XlVa(79.9);
17	Bacteria(100);Firmicutes(100);Clostridia(100);Clostridiales(100);Lachnospiraceae(97.1);Clostridium_XlVa(45.2);
18	Bacteria(100);Firmicutes(100);Clostridia(100);Clostridiales(100);Lachnospiraceae(100);Roseburia(54.7);
19	Bacteria(100);Firmicutes(100);Clostridia(100);Clostridiales(100);Lachnospiraceae(99.9);Marvinbryantia(33.5);
20	Bacteria(100);Firmicutes(100);Clostridia(100);Clostridiales(100);Ruminococcaceae(100);Oscillibacter(100);
21	Bacteria(100);Firmicutes(100);Clostridia(100);Clostridiales(100);Lachnospiraceae(99.5);Lachnospiracea_incertae_sedis(85.3);
22	Bacteria(100);Firmicutes(100);Clostridia(100);Clostridiales(100);Lachnospiraceae(97.8);Clostridium_XlVa(82.5);
23	Bacteria(100);Firmicutes(100);Clostridia(100);Clostridiales(100);Lachnospiraceae(100);Clostridium_XlVb(100);
24	Bacteria(100);Firmicutes(100);Clostridia(100);Clostridiales(100);Lachnospiraceae(100);Roseburia(73.6);
25	Bacteria(100);Firmicutes(100);Clostridia(100);Clostridiales(100);Ruminococcaceae(100);Flavonifractor(72.5);
26	Bacteria(100);Firmicutes(100);Clostridia(100);Clostridiales(100);Ruminococcaceae(100);Clostridium_IV(98.9);
27	Bacteria(100);Firmicutes(71.2);Clostridia(69.4);Clostridiales(68.1);Lachnospiraceae(36.8);Cellulosilyticum(15.1);
28	Bacteria(100);Firmicutes(100);Clostridia(100);Clostridiales(100);Lachnospiraceae(99.4);Coprococcus(28.7);
29	Bacteria(100);Firmicutes(100);Clostridia(100);Clostridiales(100);Ruminococcaceae(100);Clostridium_IV(39.7);
30	Bacteria(100);Firmicutes(100);Bacilli(100);Lactobacillales(100);Streptococcaceae(100);Lactococcus(100);
31	Bacteria(100);Firmicutes(99.7);Clostridia(99.7);Clostridiales(99.7);Lachnospiraceae(97.9);Syntrophococcus(38.7);
32	Bacteria(100);Firmicutes(100);Clostridia(100);Clostridiales(100);Lachnospiraceae(99.2);Clostridium_XlVa(47.8);
33	Bacteria(100);Firmicutes(93.2);Clostridia(90.7);Clostridiales(89.5);Gracilibacteraceae(22);Gracilibacter(21.9);
34	Bacteria(100);Firmicutes(98.2);Clostridia(96.4);Clostridiales(96.4);Ruminococcaceae(81.7);Clostridium_IV(43.8);
35	Bacteria(100);Firmicutes(55.4);Clostridia(48.9);Clostridiales(47.7);Ruminococcaceae(31.3);Butyricicoccus(20.4);
36	Bacteria(100);"Bacteroidetes"(100);"Bacteroidia"(98.1);"Bacteroidales"(98.1);"Porphyromonadaceae"(91.5);Barnesiella(51.7);
37	Bacteria(100);Firmicutes(100);Clostridia(100);Clostridiales(100);Ruminococcaceae(100);Clostridium_IV(71.7);
38	Bacteria(100);Firmicutes(100);Clostridia(100);Clostridiales(100);Lachnospiraceae(100);Hespellia(36.7);
39	Bacteria(100);Firmicutes(78.8);Clostridia(77.5);Clostridiales(77.3);Ruminococcaceae(60.4);Butyricicoccus(31.3);
40	Bacteria(100);"Bacteroidetes"(100);"Bacteroidia"(97.2);"Bacteroidales"(97.2);"Porphyromonadaceae"(80.2);Barnesiella(75.7);
41	Bacteria(100);Firmicutes(100);Clostridia(100);Clostridiales(100);Clostridiaceae_1(100);Clostridium_sensu_stricto(99.4);
42	Bacteria(100);Firmicutes(100);Clostridia(100);Clostridiales(100);Lachnospiraceae(100);Moryella(39.1);
43	Bacteria(99.9);"Bacteroidetes"(99.4);"Bacteroidia"(96.7);"Bacteroidales"(96.7);"Porphyromonadaceae"(88.4);Barnesiella(59.3);
44	Bacteria(100);Firmicutes(98.8);Clostridia(98.7);Clostridiales(98.7);Lachnospiraceae(90.2);Marvinbryantia(38.4);
45	Bacteria(100);Firmicutes(99.5);Clostridia(99.2);Clostridiales(99.2);Lachnospiraceae(91.1);Clostridium_XlVb(48.7);
46	Bacteria(100);Firmicutes(100);Clostridia(100);Clostridiales(100);Ruminococcaceae(100);Clostridium_IV(98.2);
47	Bacteria(100);Firmicutes(100);Clostridia(100);Clostridiales(100);Lachnospiraceae(99.9);Acetitomaculum(19.4);
48	Bacteria(100);Firmicutes(100);Clostridia(100);Clostridiales(99.9);Eubacteriaceae(79.3);Eubacterium(79.3);
49	Bacteria(100);"Bacteroidetes"(100);Flavobacteria(100);"Flavobacteriales"(100);Flavobacteriaceae(100);Flavobacterium(100);
50	Bacteria(100);Firmicutes(100);Clostridia(100);Clostridiales(100);Lachnospiraceae(98.4);Clostridium_XlVa(40.6);
51	Bacteria(100);Firmicutes(100);Clostridia(100);Clostridiales(100);Lachnospiraceae(99.9);Clostridium_XlVb(99.9);
52	Bacteria(100);Firmicutes(80.3);Clostridia(78.4);Clostridiales(78.2);Ruminococcaceae(55.3);Butyricicoccus(33.1);
53	Bacteria(100);Firmicutes(100);Clostridia(100);Clostridiales(100);Lachnospiraceae(100);Syntrophococcus(16);
54	Bacteria(100);Firmicutes(100);Clostridia(100);Clostridiales(100);Lachnospiraceae(100);Lachnospiracea_incertae_sedis(78.3);
55	Bacteria(100);Firmicutes(100);Clostridia(100);Clostridiales(100);Lachnospiraceae(100);Roseburia(100);
56	Bacteria(100);Firmicutes(100);Clostridia(100);Clostridiales(100);Ruminococcaceae(100);Clostridium_IV(99.4);
57	Bacteria(100);Firmicutes(99.9);Clostridia(99.9);Clostridiales(99.9);Lachnospiraceae(99.8);Roseburia(52.9);
58	Bacteria(100);Firmicutes(100);Clostridia(100);Clostridiales(100);Lachnospiraceae(97.7);Coprococcus(15.9);
59	Bacteria(100);Firmicutes(100);Clostridia(100);Clostridiales(100);Lachnospiraceae(100);Oribacterium(41.7);
60	Bacteria(100);Firmicutes(100);Clostridia(100);Clostridiales(100);Ruminococcaceae(100);Clostridium_IV(87.2);
61	Bacteria(100);Firmicutes(100);Clostridia(100);Clostridiales(100);Ruminococcaceae(100);Acetanaerobacterium(60.9);
62	Bacteria(100);Firmicutes(100);Erysipelotrichia(100);Erysipelotrichales(100);Erysipelotrichaceae(100);Clostridium_XVIII(100);
63	Bacteria(100);Firmicutes(100);Clostridia(100);Clostridiales(100);Lachnospiraceae(95.1);Butyrivibrio(16.1);
64	Bacteria(100);Firmicutes(95.7);Clostridia(94.4);Clostridiales(94.2);Ruminococcaceae(89.2);Flavonifractor(16.3);
65	Bacteria(100);Firmicutes(100);Clostridia(100);Clostridiales(100);Ruminococcaceae(100);Anaerotruncus(85.4);
66	Bacteria(100);Firmicutes(81.1);Clostridia(76.5);Clostridiales(75);Ruminococcaceae(34.7);Butyricicoccus(17.2);
67	Bacteria(100);"Tenericutes"(100);Mollicutes(100);Anaeroplasmatales(100);Anaeroplasmataceae(100);Anaeroplasma(100);
68	Bacteria(100);Firmicutes(100);Clostridia(100);Clostridiales(100);Ruminococcaceae(100);Oscillibacter(98.7);
69	Bacteria(100);Firmicutes(100);Clostridia(100);Clostridiales(100);Lachnospiraceae(89.4);Coprococcus(14.3);
70	Bacteria(100);Firmicutes(100);Clostridia(100);Clostridiales(100);Lachnospiraceae(97.5);Moryella(32.5);
71	Bacteria(100);Firmicutes(46.2);Clostridia(45.2);Clostridiales(41.7);Incertae_Sedis_IV(6.7);Caldicoprobacter(6.7);
72	Bacteria(100);Firmicutes(100);Clostridia(100);Clostridiales(100);Ruminococcaceae(100);Anaerotruncus(48.4);
73	Bacteria(100);Firmicutes(100);Clostridia(100);Clostridiales(100);Lachnospiraceae(100);Lachnospiracea_incertae_sedis(39.1);
74	Bacteria(100);Firmicutes(100);Clostridia(100);Clostridiales(100);Lachnospiraceae(93.3);Syntrophococcus(28);
75	Bacteria(100);Firmicutes(100);Clostridia(100);Clostridiales(100);Ruminococcaceae(99.7);Pseudoflavonifractor(65.3);
76	Bacteria(100);Firmicutes(100);Erysipelotrichia(100);Erysipelotrichales(100);Erysipelotrichaceae(100);Coprobacillus(61.6);
77	Bacteria(100);Firmicutes(100);Clostridia(100);Clostridiales(100);Lachnospiraceae(98.4);Lachnospiracea_incertae_sedis(22);
78	Bacteria(100);"Bacteroidetes"(100);"Sphingobacteria"(100);"Sphingobacteriales"(100);Sphingobacteriaceae(100);Pedobacter(100);
79	Bacteria(100);Firmicutes(100);Clostridia(100);Clostridiales(100);Ruminococcaceae(100);Clostridium_IV(36.2);
80	Bacteria(100);Firmicutes(100);Clostridia(100);Clostridiales(100);Lachnospiraceae(98.4);Clostridium_XlVa(40);
81	Bacteria(100);Firmicutes(100);Clostridia(100);Clostridiales(100);Lachnospiraceae(100);Clostridium_XlVa(50.3);
82	Bacteria(100);Firmicutes(75.4);Clostridia(69.9);Clostridiales(67.9);Ruminococcaceae(35.2);Acetivibrio(18);
83	Bacteria(100);Firmicutes(100);Erysipelotrichia(100);Erysipelotrichales(100);Erysipelotrichaceae(100);Erysipelotrichaceae_incertae_sedis(98.4);

84	Bacteria(100);Firmicutes(98.9);Negativicutes(66.4);Selenomonadales(66.4);Veillonellaceae(65.4);Zymophilus(23.4);
85	Bacteria(100);Firmicutes(99.8);Clostridia(99.8);Clostridiales(99.8);Ruminococcaceae(99.1);Clostridium_IV(79.5);
86	Bacteria(100);Firmicutes(100);Clostridia(100);Clostridiales(100);Lachnospiraceae(100);Lachnospiracea_incertae_sedis(88.1);
87	Bacteria(100);Firmicutes(100);Clostridia(99.9);Clostridiales(99.9);Lachnospiraceae(97.2);Marvinbryantia(54.1);
88	Bacteria(100);Firmicutes(99.9);Clostridia(99.9);Clostridiales(99.9);Lachnospiraceae(96.8);Clostridium_XlVa(29.3);
89	Bacteria(100);Firmicutes(100);Clostridia(100);Clostridiales(100);Ruminococcaceae(100);Butyricicoccus(98.4);
90	Bacteria(100);Firmicutes(100);Clostridia(100);Clostridiales(100);Lachnospiraceae(100);Clostridium_XlVa(63.8);
91	Bacteria(100);Firmicutes(100);Clostridia(100);Clostridiales(100);Lachnospiraceae(100);Blautia(43.4);
92	Bacteria(100);"Bacteroidetes"(100);"Bacteroidia"(99);"Bacteroidales"(99);"Porphyromonadaceae"(94.9);Barnesiella(65.8);
93	Bacteria(100);Firmicutes(100);Clostridia(100);Clostridiales(100);Lachnospiraceae(99.9);Clostridium_XlVa(54.1);
94	Bacteria(100);Firmicutes(100);Clostridia(100);Clostridiales(100);Lachnospiraceae(100);Clostridium_XlVa(62.3);
95	Bacteria(100);Firmicutes(100);Clostridia(100);Clostridiales(100);Lachnospiraceae(99.9);Clostridium_XlVa(52.1);
96	Bacteria(100);Firmicutes(100);Clostridia(100);Clostridiales(100);Lachnospiraceae(100);Clostridium_XlVa(73.2);
97	Bacteria(100);Firmicutes(100);Clostridia(100);Clostridiales(100);Lachnospiraceae(95.9);Lachnospiracea_incertae_sedis(40.3);
98	Bacteria(100);"Bacteroidetes"(100);"Bacteroidia"(99.9);"Bacteroidales"(99.9);"Porphyromonadaceae"(99.6);Barnesiella(89.8);
99	Bacteria(100);Firmicutes(100);Clostridia(100);Clostridiales(100);Lachnospiraceae(98.6);Clostridium_XlVa(44.4);
100	Bacteria(100);"Actinobacteria"(99.8);Actinobacteria(99.8);Coriobacteriales(99.8);Coriobacteriaceae(99.8);Paraeggerthella(48.2);
101	Bacteria(100);"Actinobacteria"(100);Actinobacteria(100);Coriobacteriales(100);Coriobacteriaceae(100);Olsenella(80.5);
102	Bacteria(100);Firmicutes(99.9);Clostridia(99.9);Clostridiales(99.9);Ruminococcaceae(96.5);Acetivibrio(53.4);
103	Bacteria(100);Firmicutes(94.6);Clostridia(91.4);Clostridiales(91.4);Ruminococcaceae(85.9);Pseudoflavonifractor(26.4);
104	Bacteria(100);Firmicutes(100);Clostridia(100);Clostridiales(100);Lachnospiraceae(99.2);Clostridium_XlVa(60.3);
105	Bacteria(100);Firmicutes(100);Clostridia(100);Clostridiales(100);Lachnospiraceae(99.8);Clostridium_XlVa(32.9);
106	Bacteria(100);Firmicutes(100);Bacilli(100);Lactobacillales(100);Lactobacillaceae(100);Lactobacillus(100);
107	Bacteria(100);Firmicutes(99.8);Clostridia(99.8);Clostridiales(99.8);Lachnospiraceae(99);Marvinbryantia(36.1);
108	Bacteria(100);Firmicutes(100);Clostridia(100);Clostridiales(100);Lachnospiraceae(99.1);Blautia(31.5);
109	Bacteria(100);Firmicutes(100);Clostridia(100);Clostridiales(100);Lachnospiraceae(100);Dorea(30.6);
110	Bacteria(100);Firmicutes(96.9);Clostridia(59.4);Clostridiales(53.2);Peptococcaceae_2(19.9);Cryptanaerobacter(19.2);
111	Bacteria(100);Firmicutes(100);Clostridia(100);Clostridiales(100);Lachnospiraceae(99.9);Clostridium_XlVa(38.3);
112	Bacteria(100);Firmicutes(100);Erysipelotrichia(100);Erysipelotrichales(100);Erysipelotrichaceae(100);Erysipelotrichaceae_incertae_sedis(100);
113	Bacteria(100);Firmicutes(100);Clostridia(100);Clostridiales(100);Lachnospiraceae(96.6);Coprococcus(43.5);
114	Bacteria(100);Firmicutes(100);Clostridia(99.2);Clostridiales(99.2);Ruminococcaceae(92.3);Acetivibrio(77.9);
115	Bacteria(100);Firmicutes(100);Bacilli(100);Lactobacillales(100);Streptococcaceae(100);Streptococcus(100);
116	Bacteria(100);"Proteobacteria"(100);Gammaproteobacteria(100);Pseudomonadales(100);Pseudomonadaceae(100);Pseudomonas(100);
117	Bacteria(100);Firmicutes(100);Bacilli(100);Lactobacillales(100);Enterococcaceae(100);Enterococcus(100);
118	Bacteria(99.9);Firmicutes(87);Erysipelotrichia(53.6);Erysipelotrichales(53.6);Erysipelotrichaceae(53.6);Coprobacillus(13.1);
119	Bacteria(100);Firmicutes(100);Clostridia(100);Clostridiales(100);Lachnospiraceae(96.8);Clostridium_XlVa(66.2);
120	Bacteria(100);Firmicutes(100);Clostridia(100);Clostridiales(100);Lachnospiraceae(100);Clostridium_XlVa(46.4);
121	Bacteria(100);Firmicutes(89.2);Clostridia(82.5);Clostridiales(78.6);Clostridiales_Incertae_Sedis_XIII(60.1);Anaerovorax(60.1);
122	Bacteria(100);"Proteobacteria"(100);Alphaproteobacteria(100);Caulobacterales(100);Caulobacteraceae(100);Brevundimonas(100);
123	Bacteria(100);Firmicutes(100);Clostridia(100);Clostridiales(100);Ruminococcaceae(100);Anaerotruncus(99.6);
124	Bacteria(100);Firmicutes(100);Clostridia(100);Clostridiales(100);Ruminococcaceae(100);Oscillibacter(94.4);
125	Bacteria(100);Firmicutes(100);Clostridia(100);Clostridiales(100);Lachnospiraceae(97.4);Clostridium_XlVa(39.4);
126	Bacteria(100);Firmicutes(84.9);Clostridia(72.1);Clostridiales(69.6);Ruminococcaceae(20.5);Acetivibrio(12.5);
127	Bacteria(100);"Actinobacteria"(100);Actinobacteria(100);Coriobacteriales(100);Coriobacteriaceae(100);Enterorhabdus(99.2);
128	Bacteria(100);Firmicutes(100);Clostridia(100);Clostridiales(100);Lachnospiraceae(100);Lachnospiracea_incertae_sedis(36.2);
129	Bacteria(100);Firmicutes(100);Clostridia(100);Clostridiales(100);Lachnospiraceae(100);Coprococcus(47.7);
130	Bacteria(100);Firmicutes(100);Clostridia(100);Clostridiales(100);Lachnospiraceae(99.9);Clostridium_XlVb(99.7);
131	Bacteria(100);Firmicutes(100);Bacilli(100);Bacillales(100);Staphylococcaceae(100);Staphylococcus(100);
132	Bacteria(100);Firmicutes(80.5);Clostridia(77.1);Clostridiales(75.6);Ruminococcaceae(48.3);Acetivibrio(22.6);
133	Bacteria(100);Firmicutes(100);Clostridia(100);Clostridiales(100);Ruminococcaceae(100);Clostridium_IV(43.8);
134	Bacteria(100);Firmicutes(100);Clostridia(100);Clostridiales(100);Ruminococcaceae(100);Flavonifractor(82.7);
135	Bacteria(100);Firmicutes(100);Clostridia(100);Clostridiales(100);Lachnospiraceae(97.1);Blautia(29.5);
136	Bacteria(100);Firmicutes(100);Clostridia(100);Clostridiales(100);Ruminococcaceae(100);Clostridium_IV(99.9);
137	Bacteria(100);"Actinobacteria"(100);Actinobacteria(100);Coriobacteriales(100);Coriobacteriaceae(100);Asaccharobacter(81.6);
138	Bacteria(100);Firmicutes(100);Clostridia(100);Clostridiales(100);Lachnospiraceae(100);Clostridium_XlVa(75.8);
139	Bacteria(100);Firmicutes(100);Clostridia(100);Clostridiales(100);Lachnospiraceae(100);Lachnospiracea_incertae_sedis(94.3);
140	Bacteria(100);Firmicutes(95);Clostridia(93.9);Clostridiales(93.5);Lachnospiraceae(64.7);Cellulosilyticum(22.2);
141	Bacteria(100);Firmicutes(100);Clostridia(100);Clostridiales(100);Lachnospiraceae(95.7);Clostridium_XlVa(31.1);
142	Bacteria(100);Firmicutes(99.9);Clostridia(99.9);Clostridiales(99.9);Eubacteriaceae(52.3);Eubacterium(52.3);
143	Bacteria(100);Firmicutes(100);Clostridia(100);Clostridiales(100);Lachnospiraceae(97.4);Clostridium_XlVa(25.4);
144	Bacteria(100);Firmicutes(100);Clostridia(100);Clostridiales(100);Ruminococcaceae(100);Clostridium_IV(97);
145	Bacteria(100);Firmicutes(100);Clostridia(100);Clostridiales(100);Lachnospiraceae(100);Butyrivibrio(23.3);
146	Bacteria(100);Firmicutes(99.4);Clostridia(99.4);Clostridiales(99.4);Ruminococcaceae(99);Hydrogenoanaerobacterium(42.5);
147	Bacteria(100);Firmicutes(100);Clostridia(100);Clostridiales(100);Ruminococcaceae(100);Clostridium_IV(94.3);
148	Bacteria(100);Firmicutes(100);Clostridia(100);Clostridiales(100);Eubacteriaceae(63.7);Eubacterium(63.7);
149	Bacteria(100);Firmicutes(99.7);Clostridia(99.7);Clostridiales(99.7);Ruminococcaceae(99.7);Clostridium_IV(53.2);
150	Bacteria(100);"Bacteroidetes"(100);"Bacteroidia"(99.1);"Bacteroidales"(99.1);"Porphyromonadaceae"(98.9);Barnesiella(95.5);
151	Bacteria(100);Firmicutes(92.9);Erysipelotrichia(74.9);Erysipelotrichales(74.9);Erysipelotrichaceae(74.9);Coprobacillus(32.5);
152	Bacteria(100);Firmicutes(84.3);Clostridia(83.2);Clostridiales(81.8);Ruminococcaceae(24.2);Butyricicoccus(15.1);
153	Bacteria(100);Firmicutes(99.5);Clostridia(89.2);Clostridiales(88.4);Peptococcaceae_1(81);Peptococcus(80.2);
154	Bacteria(100);Firmicutes(83.3);Clostridia(81.6);Clostridiales(81.3);Incertae_Sedis_IV(11.4);Caldicoprobacter(11.4);
155	Bacteria(100);Firmicutes(100);Clostridia(100);Clostridiales(100);Ruminococcaceae(100);Flavonifractor(65);
156	Bacteria(100);Firmicutes(100);Clostridia(100);Clostridiales(100);Lachnospiraceae(99.7);Lachnospiracea_incertae_sedis(50.9);
157	Bacteria(100);Firmicutes(100);Clostridia(100);Clostridiales(100);Ruminococcaceae(100);Flavonifractor(95.1);
158	Bacteria(100);Firmicutes(100);Clostridia(100);Clostridiales(100);Ruminococcaceae(99.9);Clostridium_IV(70.7);
159	Bacteria(100);Firmicutes(100);Clostridia(100);Clostridiales(100);Ruminococcaceae(99.9);Clostridium_IV(43.7);
160	Bacteria(100);Firmicutes(99.8);Erysipelotrichia(95.4);Erysipelotrichales(95.4);Erysipelotrichaceae(95.4);Turicibacter(95.4);
161	Bacteria(100);"Bacteroidetes"(66.4);"Bacteroidia"(54);"Bacteroidales"(54);"Porphyromonadaceae"(43.4);Tannerella(11.6);
162	Bacteria(100);Firmicutes(100);Clostridia(100);Clostridiales(100);Lachnospiraceae(96.3);Johnsonella(24.5);
163	Bacteria(100);Firmicutes(100);Clostridia(100);Clostridiales(100);Lachnospiraceae(100);Lachnospiracea_incertae_sedis(93);
164	Bacteria(100);Firmicutes(100);Clostridia(100);Clostridiales(100);Ruminococcaceae(100);Clostridium_IV(84.4);
165	Bacteria(100);"Proteobacteria"(100);Gammaproteobacteria(100);Xanthomonadales(100);Xanthomonadaceae(100);Stenotrophomonas(99.7);
166	Bacteria(100);Firmicutes(100);Clostridia(100);Clostridiales(100);Lachnospiraceae(99.9);Clostridium_XlVa(66.7);
167	Bacteria(100);Firmicutes(75.6);Erysipelotrichia(75.6);Erysipelotrichales(75.6);Erysipelotrichaceae(75.6);Holdemania(55.6);
168	Bacteria(100);Firmicutes(100);Clostridia(100);Clostridiales(100);Lachnospiraceae(99.1);Oribacterium(58.6);
169	Bacteria(100);Firmicutes(100);Bacilli(100);Bacillales(100);Staphylococcaceae(100);Staphylococcus(100);

170 Bacteria(100);Firmicutes(100);Clostridia(100);Clostridiales(100);Lachnospiraceae(100);Roseburia(77.7);
171 Bacteria(100);Firmicutes(100);Clostridia(100);Clostridiales(100);Lachnospiraceae(100);Clostridium_XlVa(75.2);
172 Bacteria(100);Firmicutes(100);Bacilli(100);Bacillales(100);Staphylococcaceae(100);Jeotgalicoccus(100);
173 Bacteria(100);Firmicutes(99.9);Clostridia(99.3);Clostridiales(99.3);Eubacteriaceae(63.4);Eubacterium(63.4);
174 Bacteria(99.9);"Bacteroidetes"(54.8);"Bacteroidia"(39.8);"Bacteroidales"(39.8);"Porphyromonadaceae"(36.5);Tannerella(22.9);
175 Bacteria(100);Firmicutes(94.4);Clostridia(91.3);Clostridiales(90.4);Clostridiales_Incertae_Sedis_XII(39.3);Acidaminobacter(30.5);
176 Bacteria(100);Firmicutes(100);Clostridia(100);Clostridiales(100);Lachnospiraceae(100);Lachnospiracea_incertae_sedis(55.8);
177 Bacteria(100);Firmicutes(100);Clostridia(100);Clostridiales(100);Lachnospiraceae(97);Clostridium_XlVa(66.8);
178 Bacteria(100);Firmicutes(100);Clostridia(100);Clostridiales(100);Lachnospiraceae(99.7);Clostridium_XlVa(44);
179 Bacteria(100);Firmicutes(98.4);Clostridia(98.3);Clostridiales(98.3);Ruminococcaceae(98.3);Oscillibacter(82.9);
180 Bacteria(100);"Proteobacteria"(100);Gammaproteobacteria(100);"Enterobacteriales"(100);Enterobacteriaceae(100);Cronobacter(43);
181 Bacteria(100);"Proteobacteria"(100);Alphaproteobacteria(100);Rhizobiales(100);Phyllobacteriaceae(100);Phyllobacterium(100);
182 Bacteria(100);"Actinobacteria"(100);Actinobacteria(100);Bifidobacteriales(100);Bifidobacteriaceae(100);Bifidobacterium(100);
183 Bacteria(100);Firmicutes(80.7);Clostridia(70.8);Clostridiales(70.4);Gracilibacteraceae(31.1);Gracilibacter(29.6);
184 Bacteria(100);Firmicutes(100);Bacilli(100);Bacillales(99.9);Bacillaceae_1(44.5);Bacillus(44.5);
185 Bacteria(100);Firmicutes(100);Clostridia(100);Clostridiales(100);Lachnospiraceae(99.9);Lachnospiracea_incertae_sedis(80.8);
186 Bacteria(100);Firmicutes(100);Clostridia(100);Clostridiales(100);Ruminococcaceae(100);Anaerotruncus(78.9);
187 Bacteria(100);Firmicutes(100);Clostridia(100);Clostridiales(100);Lachnospiraceae(100);Roseburia(60.2);
188 Bacteria(100);Firmicutes(100);Clostridia(100);Clostridiales(100);Lachnospiraceae(99.7);Coprococcus(35.1);
189 Bacteria(100);"Actinobacteria"(100);Actinobacteria(100);Coriobacteriales(100);Coriobacteriaceae(100);Paraeggerthella(29.4);
190 Bacteria(100);Firmicutes(100);Clostridia(100);Clostridiales(100);Lachnospiraceae(96.2);Lachnospiracea_incertae_sedis(27);
191 Bacteria(100);Firmicutes(92.9);Clostridia(92.9);Clostridiales(92.9);Ruminococcaceae(92.8);Pseudoflavonifractor(52.1);
192 Bacteria(100);Firmicutes(100);Clostridia(100);Clostridiales(100);Ruminococcaceae(100);Sporobacter(48.9);
193 Bacteria(100);Firmicutes(100);Clostridia(100);Clostridiales(100);Ruminococcaceae(100);Oscillibacter(100);
194 Bacteria(100);Firmicutes(100);Clostridia(100);Clostridiales(100);Lachnospiraceae(93.7);Clostridium_XlVa(60.2);
195 Bacteria(100);Firmicutes(100);Clostridia(100);Clostridiales(100);Ruminococcaceae(99.9);Anaerotruncus(57.9);
196 Bacteria(100);Firmicutes(94.6);Clostridia(93.4);Clostridiales(92.4);Clostridiales_Incertae_Sedis_XII(26.6);Guggenheimella(16.5);
197 Bacteria(100);Firmicutes(100);Clostridia(100);Clostridiales(100);Lachnospiraceae(92.9);Lachnospiracea_incertae_sedis(37.7);
198 Bacteria(100);"Proteobacteria"(100);Betaproteobacteria(100);Burkholderiales(100);Burkholderiaceae(100);Ralstonia(99.9);
199 Bacteria(99.9);"Bacteroidetes"(51);"Bacteroidia"(36);"Bacteroidales"(36);"Porphyromonadaceae"(30.8);Tannerella(14.1);
200 Bacteria(100);Firmicutes(100);Clostridia(100);Clostridiales(100);Lachnospiraceae(99.9);Lachnospiracea_incertae_sedis(36.4);
201 Bacteria(100);Firmicutes(100);Clostridia(100);Clostridiales(100);Lachnospiraceae(99.8);Blautia(31.8);
202 Bacteria(100);Firmicutes(77.8);Clostridia(77.7);Clostridiales(77.7);Peptostreptococcaceae(73);Sporacetigenium(56.5);
203 Bacteria(100);Firmicutes(66.3);Clostridia(64.7);Clostridiales(64.6);Ruminococcaceae(63.6);Ethanoligenens(51.8);
204 Bacteria(100);"Proteobacteria"(100);Betaproteobacteria(100);Burkholderiales(100);Comamonadaceae(100);Delftia(99.3);
205 Bacteria(100);Firmicutes(99.9);Clostridia(99.9);Clostridiales(99.9);Lachnospiraceae(97.9);Marvinbryantia(44.5);
206 Bacteria(100);"Actinobacteria"(100);Actinobacteria(100);Actinomycetales(100);Nocardiaceae(100);Rhodococcus(100);
207 Bacteria(100);"Bacteroidetes"(89.2);"Bacteroidia"(87.8);"Bacteroidales"(87.8);"Porphyromonadaceae"(75.2);Tannerella(45.5);
208 Bacteria(100);Firmicutes(92.5);Clostridia(90.2);Clostridiales(88.4);Ruminococcaceae(46.9);Clostridium_IV(32.2);
209 Bacteria(100);Firmicutes(97.2);Erysipelotrichia(96.9);Erysipelotrichales(96.9);Erysipelotrichaceae(96.9);Turicibacter(96.9);
210 Bacteria(100);Firmicutes(68.4);Clostridia(65.2);Clostridiales(65);Ruminococcaceae(31.7);Butyricicoccus(20.1);
211 Bacteria(100);"Actinobacteria"(100);Actinobacteria(100);Coriobacteriales(100);Coriobacteriaceae(100);Enterorhabdus(100);
212 Bacteria(100);Firmicutes(77);Clostridia(76.1);Clostridiales(76);Clostridiaceae_1(61);Anaerobacter(58.6);
213 Bacteria(100);Firmicutes(85.6);Clostridia(85.1);Clostridiales(84.7);Ruminococcaceae(44.2);Clostridium_IV(32.4);
214 Bacteria(100);Firmicutes(56.4);Clostridia(56.4);Clostridiales(56.4);Lachnospiraceae(56.3);Johnsonella(48.1);
215 Bacteria(100);"Proteobacteria"(100);Alphaproteobacteria(100);Rhizobiales(100);Hyphomicrobiaceae(100);Devosia(100);
216 Bacteria(100);"Bacteroidetes"(100);"Bacteroidia"(98.8);"Bacteroidales"(98.8);"Porphyromonadaceae"(95.7);Barnesiella(43.7);
217 Bacteria(100);Firmicutes(99.8);Erysipelotrichia(99.7);Erysipelotrichales(99.7);Erysipelotrichaceae(99.7);Allobaculum(85.4);
218 Bacteria(100);Firmicutes(70.4);Clostridia(69.9);Clostridiales(69.9);Ruminococcaceae(69);Anaerotruncus(37);
219 Bacteria(100);Firmicutes(100);Clostridia(100);Clostridiales(100);Lachnospiraceae(97.3);Marvinbryantia(39.2);
220 Bacteria(100);Firmicutes(100);Clostridia(100);Clostridiales(100);Lachnospiraceae(62.1);Oribacterium(10.7);
221 Bacteria(100);Firmicutes(100);Clostridia(100);Clostridiales(100);Ruminococcaceae(100);Oscillibacter(95.6);
222 Bacteria(100);"Proteobacteria"(100);Alphaproteobacteria(100);Sphingomonadales(100);Sphingomonadaceae(100);Sphingomonas(99.9);
223 Bacteria(99.9);Firmicutes(96.7);Clostridia(96.5);Clostridiales(96.5);Lachnospiraceae(86.5);Robinsoniella(14.5);
224 Bacteria(100);Firmicutes(100);Clostridia(100);Clostridiales(100);Lachnospiraceae(99.9);Butyrivibrio(49);
225 Bacteria(100);"Proteobacteria"(100);Gammaproteobacteria(100);Xanthomonadales(100);Xanthomonadaceae(100);Stenotrophomonas(94.6);
226 Bacteria(100);Firmicutes(100);Clostridia(100);Clostridiales(100);Lachnospiraceae(99.9);Lachnobacterium(24);
227 Bacteria(100);Firmicutes(100);Bacilli(100);Lactobacillales(100);Streptococcaceae(100);Streptococcus(100);
228 Bacteria(100);Firmicutes(100);Clostridia(100);Clostridiales(100);Lachnospiraceae(100);Lachnospiracea_incertae_sedis(47.6);
229 Bacteria(100);"Actinobacteria"(100);Actinobacteria(100);Coriobacteriales(100);Coriobacteriaceae(100);Adlercreutzia(4.6);
230 Bacteria(100);"Bacteroidetes"(100);"Bacteroidia"(100);"Bacteroidales"(100);Bacteroidaceae(100);Bacteroides(100);
231 Bacteria(100);Firmicutes(99.8);Bacilli(96.5);Lactobacillales(96.4);Streptococcaceae(96.3);Lactococcus(94.9);
232 Bacteria(100);Firmicutes(100);Clostridia(100);Clostridiales(100);Lachnospiraceae(99.9);Lachnospiracea_incertae_sedis(66.7);
233 Bacteria(100);"Proteobacteria"(100);Alphaproteobacteria(100);Rhizobiales(100);Rhizobiaceae(100);Rhizobium(100);
234 Bacteria(100);Firmicutes(100);Clostridia(100);Clostridiales(100);Lachnospiraceae(94.7);Syntrophococcus(35);
235 Bacteria(100);Firmicutes(59.8);Clostridia(59.3);Clostridiales(59.3);Clostridiaceae_1(52.2);Anaerobacter(49);
236 Bacteria(100);"Bacteroidetes"(100);"Bacteroidia"(100);"Bacteroidales"(100);"Prevotellaceae"(100);Prevotella(92.1);
237 Bacteria(100);"Bacteroidetes"(86.8);"Bacteroidia"(60.8);"Bacteroidales"(60.8);"Porphyromonadaceae"(52.9);Tannerella(22.4);
238 Bacteria(100);Firmicutes(100);Clostridia(100);Clostridiales(100);Ruminococcaceae(100);Flavonifractor(63);
239 Bacteria(100);Firmicutes(100);Clostridia(100);Clostridiales(100);Ruminococcaceae(100);Flavonifractor(59.1);
240 Bacteria(100);Firmicutes(99.7);Bacilli(75.8);Lactobacillales(72.7);Enterococcaceae(17.3);Melissococcus(15.2);
241 Bacteria(100);"Proteobacteria"(100);Betaproteobacteria(100);Burkholderiales(100);Alcaligenaceae(100);Achromobacter(97.4);
242 Bacteria(100);"Bacteroidetes"(100);"Bacteroidia"(100);"Bacteroidales"(100);"Porphyromonadaceae"(100);Parabacteroides(100);
243 Bacteria(100);"Actinobacteria"(100);Actinobacteria(100);Actinomycetales(100);Propionibacteriaceae(100);Propionibacterium(100);
244 Bacteria(100);"Bacteroidetes"(100);"Sphingobacteria"(100);"Sphingobacteriales"(100);Sphingobacteriaceae(100);Sphingobacterium(100);
245 Bacteria(100);Firmicutes(99.9);Clostridia(99.9);Clostridiales(99.9);Lachnospiraceae(97);Clostridium_XlVa(56.7);
246 Bacteria(100);Firmicutes(98.9);Clostridia(98.9);Clostridiales(98.9);Ruminococcaceae(98.6);Clostridium_IV(81.6);
247 Bacteria(100);Firmicutes(85.9);Clostridia(85.9);Clostridiales(85.9);Lachnospiraceae(82.5);Syntrophococcus(22.9);
248 Bacteria(100);"Bacteroidetes"(97.8);"Bacteroidia"(92.8);"Bacteroidales"(92.8);"Porphyromonadaceae"(84.9);Tannerella(37.6);
249 Bacteria(100);Firmicutes(92.2);Clostridia(87);Clostridiales(79.9);Ruminococcaceae(56.8);Saccharofermentans(21.6);
250 Eukaryota(11.9);Streptophyta(9.9);Liliopsida(9.9);Poales(9.9);Poaceae(9.9);Zea(9.9);
251 Bacteria(100);Firmicutes(100);Clostridia(94);Clostridiales(94);Ruminococcaceae(94);Flavonifractor(84.9);
252 Bacteria(87.6);"Elusimicrobia"(4.1);Elusimicrobia(4.1);Elusimicrobiales(4.1);Elusimicrobiaceae(4.1);Elusimicrobium(4.1);
253 Bacteria(100);Firmicutes(83.7);Clostridia(83.7);Clostridiales(83.7);Ruminococcaceae(83.7);Oscillibacter(64.1);
254 Bacteria(100);"Bacteroidetes"(75.4);"Bacteroidia"(70.5);"Bacteroidales"(70.5);"Porphyromonadaceae"(64.9);Tannerella(49.9);
255 Bacteria(100);"Proteobacteria"(100);Alphaproteobacteria(100);Rhizobiales(100);Phyllobacteriaceae(100);Mesorhizobium(99.3);

256 Bacteria(100);Firmicutes(95.3);Clostridia(95.3);Clostridiales(95.3);Ruminococcaceae(95.2);Flavonifractor(57.8);
257 Bacteria(86.5);"Elusimicrobia"(3.9);Elusimicrobia(3.9);Elusimicrobiales(3.9);Elusimicrobiaceae(3.9);Elusimicrobium(3.9);
258 Bacteria(100);Firmicutes(80.2);Clostridia(80);Clostridiales(80);Peptostreptococcaceae(79.5);Sporacetigenium(72.1);
259 Bacteria(100);"Bacteroidetes"(100);"Bacteroidia"(100);"Bacteroidales"(100);Bacteroidaceae(100);Bacteroides(100);
260 Bacteria(100);Firmicutes(83.8);Clostridia(83.8);Clostridiales(83.5);Lachnospiraceae(82);Clostridium_XlVb(81.8);
261 Bacteria(100);Firmicutes(86.2);Clostridia(78.7);Clostridiales(78.5);Gracilibacteraceae(32.3);Gracilibacter(30);
262 Bacteria(100);"Bacteroidetes"(100);"Bacteroidia"(98.1);"Bacteroidales"(98.1);"Porphyromonadaceae"(97.7);Barnesiella(90.3);
263 Bacteria(88.1);"Bacteroidetes"(35.1);"Bacteroidia"(12);"Bacteroidales"(12);"Porphyromonadaceae"(6);Petrimonas(4.4);
264 Bacteria(87.6);"Bacteroidetes"(22.6);Flavobacteria(5.8);"Flavobacteriales"(5.8);Flavobacteriaceae(5.1);Ornithobacterium(1.5);
265 Bacteria(100);Firmicutes(100);Clostridia(100);Clostridiales(100);Lachnospiraceae(99.9);Lachnospiracea_incertae_sedis(38.7);
266 Eukaryota(12.3);Arthropoda(8.5);Insecta(8.5);Coleoptera(8.5);Tenebrionidae(8.5);Tribolium(8.5);
267 Bacteria(100);"Bacteroidetes"(100);"Bacteroidia"(100);"Bacteroidales"(100);Bacteroidaceae(100);Bacteroides(100);
268 Bacteria(100);Firmicutes(62.9);Clostridia(62.9);Clostridiales(62.9);Ruminococcaceae(62.8);Anaerotruncus(54.9);
269 Eukaryota(13);Streptophyta(11.1);Liliopsida(11.1);Poales(11.1);Poaceae(11.1);Zea(11.1);
270 Archaea(17.5);"Euryarchaeota"(11.7);Thermoplasmata(6.6);Thermoplasmatales(6.6);Thermoplasmatales_incertae_sedis(6.4);Thermogymnomonas(6.4);
271 Bacteria(100);Firmicutes(100);Bacilli(100);Lactobacillales(100);Carnobacteriaceae(100);Atopostipes(99.6);
272 Eukaryota(28.2);Chordata(15.9);Mammalia(15.9);Primates(2.8);Hominidae(2.8);Homo(2.8);
273 Bacteria(88.8);"Bacteroidetes"(24.7);"Sphingobacteria"(14.4);"Sphingobacteriales"(14.4);Sphingobacteriaceae(3.6);Pseudosphingobacterium(2.7);
274 Eukaryota(28.9);Streptophyta(19.7);Liliopsida(19.7);Poales(19.7);Poaceae(19.7);Zea(19.7);
275 Bacteria(100);Firmicutes(96.8);Erysipelotrichia(94);Erysipelotrichales(94);Erysipelotrichaceae(94);Holdemania(49.7);
276 Eukaryota(13.1);Streptophyta(4.9);Liliopsida(4.9);Poales(4.9);Poaceae(4.9);Zea(4.9);
277 Eukaryota(23.1);Chordata(13.3);Mammalia(13.3);Primates(3.8);Hominidae(3.8);Homo(3.8);
278 Bacteria(80.8);Firmicutes(23.5);Clostridia(20);Clostridiales(16.4);Clostridiales_Incertae_Sedis_XI(7.7);Parvimonas(7.5);
279 Bacteria(100);Firmicutes(99.8);Clostridia(99.3);Clostridiales(99.3);Lachnospiraceae(63);Lactonifactor(15.5);
280 Bacteria(100);Firmicutes(95.4);Clostridia(94.8);Clostridiales(94.8);Ruminococcaceae(44.9);Saccharofermentans(22.1);
281 Bacteria(100);Firmicutes(64.3);Erysipelotrichia(63.8);Erysipelotrichales(63.8);Erysipelotrichaceae(63.8);Holdemania(49.6);
282 Bacteria(100);Firmicutes(99.5);Clostridia(99.5);Clostridiales(99.5);Ruminococcaceae(99.5);Oscillibacter(92.8);
283 Eukaryota(8.1);Streptophyta(7.1);Liliopsida(7.1);Poales(7.1);Poaceae(7.1);Zea(7.1);
284 Bacteria(78.9);"Proteobacteria"(29);Deltaproteobacteria(15.6);Myxococcales(7.8);Nannocystaceae(7.5);Enhygromyxa(7.1);
285 Bacteria(100);"Tenericutes"(76.7);Mollicutes(76.7);Anaeroplasmatales(76);Anaeroplasmataceae(76);Anaeroplasma(76);
286 Bacteria(100);"Proteobacteria"(90.1);Alphaproteobacteria(90.1);Rhizobiales(90.1);Hyphomicrobiaceae(90.1);Gemmiger(90.1);
287 Bacteria(100);Firmicutes(89.4);Bacilli(89.3);Lactobacillales(87.1);Streptococcaceae(86.3);Lactococcus(82.5);
288 Bacteria(100);Firmicutes(76.1);Clostridia(71.7);Clostridiales(69.8);Ruminococcaceae(22.3);Butyricicoccus(10.3);
289 Eukaryota(16.8);Streptophyta(13.7);Liliopsida(13.7);Poales(13.7);Poaceae(13.7);Zea(13.7);
290 Bacteria(100);Firmicutes(100);Clostridia(100);Clostridiales(100);Lachnospiraceae(99.9);Oribacterium(26.4);
291 Bacteria(100);"Bacteroidetes"(100);"Bacteroidia"(100);"Bacteroidales"(100);"Prevotellaceae"(100);Prevotella(85.1);
292 Bacteria(87.5);Firmicutes(28.7);Clostridia(22.2);Clostridiales(20.2);Clostridiales_Incertae_Sedis_XI(4.4);Parvimonas(3.4);
293 Bacteria(88.1);"Proteobacteria"(39.2);Gammaproteobacteria(11.8);Legionellales(4.5);Coxiellaceae(4.5);Coxiella(4.5);
294 Bacteria(82.8);"Bacteroidetes"(27.7);"Sphingobacteria"(15.2);"Sphingobacteriales"(15.2);Cytophagaceae(13.3);Litoribacter(5.2);
295 Bacteria(100);"Spirochaetes"(96.8);Spirochaetes(96.8);Spirochaetales(96.8);Spirochaetaceae(96);Spirochaeta(92.6);
296 Archaea(22.1);"Euryarchaeota"(16.4);Halobacteria(11.9);Halobacteriales(11.9);Halobacteriaceae(11.9);Halonotius(5.1);
297 Bacteria(100);Firmicutes(64.5);Clostridia(62.2);Clostridiales(61.1);Ruminococcaceae(20.3);Butyricicoccus(12.4);
298 Eukaryota(13.6);Chordata(12.3);Mammalia(12.3);Rodentia(11.4);Muridae(11.4);Mus(11.4);
299 Eukaryota(22.2);Chordata(15.4);Mammalia(15.4);Primates(3.8);Hominidae(3.8);Homo(3.8);
300 Eukaryota(12.8);Streptophyta(11.5);Liliopsida(11.5);Poales(11.5);Poaceae(11.5);Zea(11.5);
301 Bacteria(90.1);"Deferribacteres"(6.9);Deferribacteres(6.9);Deferribacterales(6.9);Deferribacteraceae(6.9);Mucispirillum(6.8);
302 Bacteria(100);"Proteobacteria"(100);Alphaproteobacteria(100);Rhizobiales(100);Bradyrhizobiaceae(100);Bradyrhizobium(98.7);
303 Bacteria(100);Firmicutes(100);Clostridia(100);Clostridiales(100);Lachnospiraceae(100);Dorea(76.1);
304 Eukaryota(12.2);Streptophyta(10.7);Liliopsida(10.7);Poales(10.7);Poaceae(10.7);Zea(10.7);
305 Eukaryota(23.3);Chordata(21.6);Mammalia(21.6);Rodentia(15.8);Muridae(15.8);Mus(15.8);
306 Bacteria(100);Firmicutes(99.6);Clostridia(99.3);Clostridiales(99.3);Lachnospiraceae(97.1);Marvinbryantia(78.8);
307 Bacteria(88.1);"Bacteroidetes"(31.2);"Bacteroidia"(13.4);"Bacteroidales"(13.4);"Porphyromonadaceae"(7.6);Proteiniphilum(3.9);
308 Bacteria(86.5);Firmicutes(26.4);Clostridia(20.9);Clostridiales(20.1);Clostridiales_Incertae_Sedis_XI(6.3);Parvimonas(4.5);
309 Bacteria(100);Firmicutes(100);Negativicutes(100);Selenomonadales(100);Acidaminococcaceae(100);Phascolarctobacterium(100);
310 Bacteria(100);Firmicutes(100);Erysipelotrichia(100);Erysipelotrichales(100);Erysipelotrichaceae(100);Clostridium_XVIII(100);
311 Bacteria(100);Firmicutes(99.9);Clostridia(99.9);Clostridiales(99.9);Ruminococcaceae(99.9);Oscillibacter(82.2);
312 Bacteria(91.9);Firmicutes(18.4);Bacilli(6.4);Lactobacillales(5.4);Enterococcaceae(4.9);Pilibacter(4.5);
313 Bacteria(84.8);"Proteobacteria"(28.1);Alphaproteobacteria(11.6);Caulobacterales(5.8);Hyphomonadaceae(5.8);Woodsholea(4.7);
314 Bacteria(100);Firmicutes(100);Clostridia(50.1);Clostridiales(50.1);Lachnospiraceae(50);Johnsonella(44);
315 Eukaryota(4.7);Chordata(3.9);Mammalia(3.9);Rodentia(3.9);Muridae(3.9);Mus(3.9);
316 Bacteria(100);"Proteobacteria"(100);Gammaproteobacteria(100);"Enterobacteriales"(100);Enterobacteriaceae(100);Klebsiella(98.8);
317 Bacteria(100);"Bacteroidetes"(99.9);"Bacteroidia"(95);"Bacteroidales"(95);"Porphyromonadaceae"(89.9);Barnesiella(49.2);
318 Bacteria(100);Firmicutes(95.2);Clostridia(95.2);Clostridiales(95.2);Lachnospiraceae(88.5);Robinsoniella(34);
319 Bacteria(100);Firmicutes(58.7);Bacilli(58.4);Lactobacillales(55.3);Carnobacteriaceae(40.1);Desemzia(16.8);
320 Bacteria(100);Firmicutes(100);Clostridia(100);Clostridiales(100);Lachnospiraceae(99.3);Clostridium_XlVa(78.4);
321 Bacteria(99.9);"Tenericutes"(58);Mollicutes(58);Anaeroplasmatales(57.8);Anaeroplasmataceae(57.8);Asteroleplasma(57.8);
322 Bacteria(100);"Bacteroidetes"(14.1);"Bacteroidia"(13.9);"Bacteroidales"(13.9);Bacteroidaceae(13.9);Anaerorhabdus(13.9);
323 Eukaryota(12.4);Streptophyta(11.9);Liliopsida(11.9);Poales(11.9);Poaceae(11.9);Zea(11.9);
324 Eukaryota(28);Chordata(27.6);Mammalia(27.6);Rodentia(21.6);Muridae(21.6);Mus(21.6);
325 Eukaryota(11.7);Chordata(8.4);Mammalia(8.4);Rodentia(8.4);Muridae(8.4);Mus(8.4);
326 Archaea(22.3);"Euryarchaeota"(18.8);Halobacteria(10.1);Halobacteriales(10.1);Halobacteriaceae(10.1);Halonotius(5.5);
327 Eukaryota(12.1);Arthropoda(7.6);Insecta(7.6);Coleoptera(7.6);Tenebrionidae(7.6);Tribolium(7.6);
328 Eukaryota(19.4);Streptophyta(13.4);Liliopsida(13.4);Poales(13.4);Poaceae(13.4);Zea(13.4);
329 Eukaryota(24.3);Chordata(12.3);Mammalia(12.3);Rodentia(12.3);Muridae(12.3);Mus(12.3);
330 Bacteria(90);Firmicutes(26);Clostridia(20.8);Clostridiales(19.1);Clostridiales_Incertae_Sedis_XI(4.2);Parvimonas(3);
331 Eukaryota(9.6);Streptophyta(8);Liliopsida(8);Poales(8);Poaceae(8);Zea(8);
332 Bacteria(87.2);"Bacteroidetes"(30.8);"Bacteroidia"(11.3);"Bacteroidales"(11.3);"Porphyromonadaceae"(6.9);Petrimonas(4.1);
333 Bacteria(100);Firmicutes(100);Erysipelotrichia(100);Erysipelotrichales(100);Erysipelotrichaceae(100);Turicibacter(100);
334 Bacteria(100);Firmicutes(100);Negativicutes(100);Selenomonadales(100);Veillonellaceae(100);Veillonella(100);
335 Bacteria(85.3);Firmicutes(19.9);Erysipelotrichia(7.8);Erysipelotrichales(7.8);Erysipelotrichaceae(7.8);Solobacterium(7.7);
336 Eukaryota(9.8);Streptophyta(8.4);Liliopsida(8.4);Poales(8.4);Poaceae(8.4);Zea(8.4);
337 Bacteria(100);"Tenericutes"(91.9);Mollicutes(91.9);Anaeroplasmatales(91.9);Anaeroplasmataceae(91.9);Anaeroplasma(91.9);
338 Eukaryota(18.4);Chordata(7.2);Mammalia(7.2);Primates(2.3);Hominidae(2.3);Homo(2.3);
339 Bacteria(100);Firmicutes(100);Clostridia(99.9);Clostridiales(99.9);Ruminococcaceae(87.4);Oscillibacter(86.7);
340 Bacteria(90.4);"Proteobacteria"(30.5);Alphaproteobacteria(14.6);Rhizobiales(5.2);Bradyrhizobiaceae(3.2);Agromonas(3);
341 Bacteria(84.4);"Bacteroidetes"(32);"Bacteroidia"(11.7);"Bacteroidales"(11.7);"Porphyromonadaceae"(6.4);Petrimonas(5);

342 Bacteria(86.6);"Bacteroidetes"(33.4);"Bacteroidia"(13.7);"Bacteroidales"(13.7);"Porphyromonadaceae"(6.9);Petrimonas(2.6);
343 Bacteria(87.2);"Proteobacteria"(24.8);Deltaproteobacteria(13.9);Desulfovibrionales(5.6);Desulfovibrionaceae(5.3);Bilophila(4.5);
344 Bacteria(100);Firmicutes(100);Clostridia(100);Clostridiales(100);Lachnospiraceae(99.8);Lachnospiracea_incertae_sedis(54.7);
345 Eukaryota(11.3);Streptophyta(10.2);Liliopsida(10.2);Poales(10.2);Poaceae(10.2);Zea(10.2);
346 Bacteria(100);Firmicutes(100);Clostridia(100);Clostridiales(100);Ruminococcaceae(100);Oscillibacter(99.4);
347 Bacteria(100);Firmicutes(99.7);Clostridia(99.7);Clostridiales(99.7);Lachnospiraceae(97.7);Johnsonella(38.9);
348 Eukaryota(18);Chordata(7.6);Mammalia(7.6);Primates(1.7);Hominidae(1.7);Homo(1.7);
349 Bacteria(100);Firmicutes(100);Clostridia(100);Clostridiales(100);Ruminococcaceae(100);Pseudoflavonifractor(64.8);
350 Bacteria(100);Firmicutes(99.5);Clostridia(99.5);Clostridiales(99.5);Lachnospiraceae(95.4);Acetitomaculum(30);
351 Bacteria(100);Firmicutes(43.2);Erysipelotrichia(43);Erysipelotrichales(43);Erysipelotrichaceae(43);Turicibacter(43);
352 Bacteria(100);Firmicutes(100);Clostridia(100);Clostridiales(100);Lachnospiraceae(96);Lachnospiracea_incertae_sedis(26.8);
353 Bacteria(100);Firmicutes(100);Clostridia(100);Clostridiales(100);Lachnospiraceae(100);Clostridium_XlVa(36.6);
354 Bacteria(81.5);"Proteobacteria"(36.1);Gammaproteobacteria(23.5);Chromatiales(8.8);Halothiobacillaceae(7.8);Thioalkalibacter(7.8);
355 Bacteria(100);Firmicutes(100);Clostridia(100);Clostridiales(100);Lachnospiraceae(99.3);Clostridium_XlVa(53.1);
356 Bacteria(100);"Bacteroidetes"(69.6);"Bacteroidia"(64.8);"Bacteroidales"(64.8);"Porphyromonadaceae"(61.5);Tannerella(27.9);
357 Bacteria(100);Firmicutes(77.5);Clostridia(77.3);Clostridiales(77.3);Lachnospiraceae(75.7);Lachnobacterium(47.9);
358 Bacteria(86.7);"Actinobacteria"(10.4);Actinobacteria(10.4);Actinomycetales(9.1);Micromonosporaceae(7.1);Catelliglobosispora(6.4);
359 Bacteria(100);Firmicutes(100);Erysipelotrichia(100);Erysipelotrichales(100);Erysipelotrichaceae(100);Clostridium_XVIII(100);
360 Bacteria(100);Firmicutes(100);Clostridia(100);Clostridiales(100);Lachnospiraceae(99.5);Marvinbryantia(35.4);
361 Bacteria(100);Firmicutes(100);Clostridia(100);Clostridiales(100);Lachnospiraceae(94);Clostridium_XlVa(42.2);
362 Bacteria(100);Firmicutes(100);Bacilli(100);Lactobacillales(100);Streptococcaceae(100);Streptococcus(100);
363 Eukaryota(13.2);Streptophyta(11);Liliopsida(11);Poales(11);Poaceae(11);Zea(11);
364 Bacteria(100);Firmicutes(74.8);Clostridia(74.8);Clostridiales(74.8);Lachnospiraceae(73.7);Lachnobacterium(23.8);
365 Eukaryota(10.1);Streptophyta(8.2);Liliopsida(8.2);Poales(8.2);Poaceae(8.2);Zea(8.2);
366 Eukaryota(15.7);Chordata(7.2);Mammalia(7.2);Primates(2);Hominidae(2);Homo(2);
367 Bacteria(87.8);Firmicutes(31);Clostridia(26.4);Clostridiales(24.7);Clostridiales_Incertae_Sedis_XI(5.3);Parvimonas(4.2);
368 Eukaryota(13.8);Chordata(5.8);Mammalia(5.8);Rodentia(5.8);Muridae(5.8);Mus(5.8);
369 Archaea(26.2);"Euryarchaeota"(23.6);Halobacteria(12.3);Halobacteriales(12.3);Halobacteriaceae(12.3);Halonotius(4);
370 Archaea(20.5);"Euryarchaeota"(17.5);"Methanomicrobia"(7.9);Methanosarcinales(6);Methanosarcinaceae(5.7);Methanimicrococcus(5.6);
371 Archaea(25.1);"Euryarchaeota"(21.8);Halobacteria(11.3);Halobacteriales(11.3);Halobacteriaceae(11.3);Halonotius(3.5);
372 Bacteria(100);"Bacteroidetes"(100);"Bacteroidia"(100);"Bacteroidales"(100);"Porphyromonadaceae"(100);Barnesiella(100);
373 Bacteria(89.4);"Deferribacteres"(7.4);Deferribacteres(7.4);Deferribacterales(7.4);Deferribacteraceae(7.4);Mucispirillum(6.7);
374 Bacteria(84.3);"Proteobacteria"(38.4);Alphaproteobacteria(13.9);Rhodobacterales(9.8);Rhodobacteraceae(9.8);Antarctobacter(0.9);
375 Bacteria(100);Firmicutes(100);Erysipelotrichia(77.5);Erysipelotrichales(77.5);Erysipelotrichaceae(77.5);Turicibacter(77.4);
376 Bacteria(100);Firmicutes(99.6);Clostridia(99.3);Clostridiales(99.3);Ruminococcaceae(91.1);Butyricicoccus(87.6);
377 Bacteria(100);Firmicutes(99.1);Clostridia(99.1);Clostridiales(99.1);Lachnospiraceae(90.9);Syntrophococcus(18.7);
378 Bacteria(100);"Bacteroidetes"(91.6);Flavobacteria(91.5);"Flavobacteriales"(91.5);Flavobacteriaceae(90.8);Dokdonia(38);
379 Eukaryota(25.4);Chordata(15.6);Mammalia(15.6);Rodentia(15.6);Muridae(15.6);Mus(15.6);
380 Bacteria(100);Firmicutes(97.6);Clostridia(96.9);Clostridiales(96.7);Ruminococcaceae(35.8);Anaerotruncus(30.2);
381 Bacteria(100);Firmicutes(100);Clostridia(100);Clostridiales(100);Lachnospiraceae(100);Coprococcus(32.4);
382 Eukaryota(12.4);Streptophyta(11.2);Liliopsida(11.2);Poales(11.2);Poaceae(11.2);Zea(11.2);
383 Bacteria(100);"Bacteroidetes"(56.9);Flavobacteria(13.6);"Flavobacteriales"(13.6);Cryomorphaceae(12.4);Fluviicola(12.4);
384 Bacteria(100);Firmicutes(97.4);Clostridia(96.2);Clostridiales(96.1);Ruminococcaceae(90.5);Pseudoflavonifractor(52.2);
385 Bacteria(100);Firmicutes(100);Clostridia(100);Clostridiales(100);Ruminococcaceae(100);Oscillibacter(97);
386 Bacteria(82.6);"Proteobacteria"(14.2);Deltaproteobacteria(7.1);Syntrophobacterales(6.2);Syntrophobacteraceae(6.2);Thermodesulforhabdus(6.2);
387 Eukaryota(25.1);Chordata(24.3);Mammalia(24.3);Rodentia(18.1);Muridae(18.1);Mus(18.1);
388 Bacteria(100);Firmicutes(99.5);Clostridia(99.5);Clostridiales(99.5);Ruminococcaceae(99.5);Faecalibacterium(93.1);
389 Bacteria(86);"Bacteroidetes"(35.5);"Bacteroidia"(16.8);"Bacteroidales"(16.8);"Porphyromonadaceae"(8.3);Petrimonas(3.7);
390 Bacteria(100);Firmicutes(94.8);Erysipelotrichia(70.7);Erysipelotrichales(70.7);Erysipelotrichaceae(70.7);Turicibacter(70.5);
391 Eukaryota(14.5);Chordata(10.2);Mammalia(10.2);Rodentia(10.2);Muridae(10.2);Mus(10.2);
392 Eukaryota(29.3);Chordata(25.2);Mammalia(25.2);Rodentia(19.7);Muridae(19.7);Mus(19.7);
393 Bacteria(100);Firmicutes(62.4);Clostridia(62.3);Clostridiales(62.3);Lachnospiraceae(56.8);Lactonifactor(31.8);
394 Eukaryota(12.2);Chordata(5.8);Mammalia(5.8);Primates(2.2);Hominidae(2.2);Homo(2.2);
395 Bacteria(100);Firmicutes(100);Clostridia(100);Clostridiales(100);Lachnospiraceae(99.9);Lachnospiracea_incertae_sedis(93.9);
396 Bacteria(100);Firmicutes(85.3);Clostridia(83.7);Clostridiales(83.6);Lachnospiraceae(76);Clostridium_XlVb(70.7);
397 Bacteria(100);Firmicutes(82.5);Clostridia(82.5);Clostridiales(82.5);Lachnospiraceae(78.3);Oribacterium(32.9);
398 Bacteria(100);"Bacteroidetes"(99.9);"Bacteroidia"(91.1);"Bacteroidales"(91.1);"Porphyromonadaceae"(74.1);Barnesiella(53.9);
399 Bacteria(87.5);"Bacteroidetes"(31.1);"Bacteroidia"(12.6);"Bacteroidales"(12.6);"Porphyromonadaceae"(5.7);Petrimonas(3.3);
400 Bacteria(100);"Bacteroidetes"(99.9);"Bacteroidia"(94.5);"Bacteroidales"(94.5);"Porphyromonadaceae"(91.8);Barnesiella(72.1);
401 Eukaryota(5.4);Streptophyta(4.7);Liliopsida(4.7);Poales(4.7);Poaceae(4.7);Zea(4.7);
402 Bacteria(100);Firmicutes(99.9);Clostridia(99.9);Clostridiales(99.9);Ruminococcaceae(99.8);Oscillibacter(81.9);
403 Bacteria(100);Firmicutes(83.5);Clostridia(78.1);Clostridiales(77.5);Clostridiales_Incertae_Sedis_XII(34.1);Acidaminobacter(17.7);
404 Bacteria(100);"Actinobacteria"(100);Actinobacteria(100);Actinomycetales(100);Microbacteriaceae(100);Microbacterium(100);
405 Bacteria(100);Firmicutes(100);Clostridia(100);Clostridiales(100);Lachnospiraceae(100);Clostridium_XlVa(75.6);
406 Bacteria(100);Firmicutes(79.6);Clostridia(67.4);Clostridiales(67);Ruminococcaceae(35.1);Saccharofermentans(19.1);
407 Bacteria(100);"Bacteroidetes"(100);"Bacteroidia"(98.9);"Bacteroidales"(98.9);"Porphyromonadaceae"(97.1);Porphyromonas(46.5);
408 Eukaryota(15.3);Streptophyta(12);Liliopsida(12);Poales(12);Poaceae(12);Zea(12);
409 Bacteria(100);Firmicutes(90.9);Clostridia(74);Clostridiales(70.7);Ruminococcaceae(31.4);Saccharofermentans(21.4);
410 Bacteria(100);Firmicutes(99.1);Clostridia(99.1);Clostridiales(99.1);Ruminococcaceae(98.9);Flavonifractor(74.2);
411 Bacteria(100);Firmicutes(72.4);Clostridia(72.4);Clostridiales(72.4);Clostridiaceae_1(71);Sarcina(70);
412 Archaea(16.5);"Euryarchaeota"(15.2);Halobacteria(11.3);Halobacteriales(11.3);Halobacteriaceae(11.3);Halolamina(3);
413 Bacteria(87.6);"Deferribacteres"(6.7);Deferribacteres(6.7);Deferribacterales(6.7);Deferribacteraceae(6.7);Mucispirillum(6.2);
414 Bacteria(100);Firmicutes(73);Clostridia(52);Clostridiales(51.4);Gracilibacteraceae(15.7);Gracilibacter(14.8);
415 Eukaryota(11);Arthropoda(5.4);Insecta(5.4);Coleoptera(5.4);Tenebrionidae(5.4);Tribolium(5.4);
416 Eukaryota(7.5);Arthropoda(5.2);Insecta(5.2);Coleoptera(5.2);Tenebrionidae(5.2);Tribolium(5.2);
417 Bacteria(100);Firmicutes(99.9);Clostridia(99.9);Clostridiales(99.9);Ruminococcaceae(66.1);Oscillibacter(54.8);
418 Bacteria(87.7);"Proteobacteria"(27.2);Deltaproteobacteria(9.4);Desulfovibrionales(2.5);Desulfovibrionaceae(1.7);Lawsonia(1.7);
419 Bacteria(90.6);Firmicutes(19.1);Bacilli(7.6);Lactobacillales(6.3);Enterococcaceae(5.6);Pilibacter(5.5);
420 Eukaryota(24.6);Chordata(13);Mammalia(13);Primates(2.7);Hominidae(2.7);Homo(2.7);
421 Bacteria(100);Firmicutes(91.2);Erysipelotrichia(68.8);Erysipelotrichales(68.8);Erysipelotrichaceae(68.8);Coprobacillus(25.7);
422 Eukaryota(18.5);Streptophyta(14.1);Liliopsida(14.1);Poales(14.1);Poaceae(14.1);Zea(14.1);
423 Bacteria(100);Firmicutes(89.1);Clostridia(89.1);Clostridiales(89.1);Lachnospiraceae(85.8);Marvinbryantia(40.1);
424 Bacteria(86.5);"Proteobacteria"(42);Gammaproteobacteria(22.6);Chromatiales(10.8);Halothiobacillaceae(10.4);Thioalkalibacter(10.2);
425 Eukaryota(24.7);Chordata(24.2);Mammalia(24.2);Rodentia(18.1);Muridae(18.1);Mus(18.1);
426 Bacteria(100);Firmicutes(100);Clostridia(100);Clostridiales(100);Lachnospiraceae(97);Coprococcus(30.2);
427 Archaea(18.7);"Euryarchaeota"(15.9);Halobacteria(10.4);Halobacteriales(10.4);Halobacteriaceae(10.4);Halonotius(2.4);

428 Bacteria(100);Firmicutes(99.4);Clostridia(96.1);Clostridiales(96);Peptostreptococcaceae(74.1);Sporacetigenium(45.1);
429 Bacteria(100);Firmicutes(77.3);Clostridia(74.7);Clostridiales(74.5);Lachnospiraceae(44.6);Lactonifactor(13.7);
430 Bacteria(100);Firmicutes(36.4);Clostridia(36.4);Clostridiales(36.4);Clostridiaceae_1(35);Sarcina(34.4);
431 Bacteria(100);Firmicutes(99.6);Erysipelotrichia(52.8);Erysipelotrichales(52.8);Erysipelotrichaceae(52.8);Turicibacter(51.5);
432 Eukaryota(11);Chordata(6.6);Mammalia(6.6);Rodentia(6.6);Muridae(6.6);Mus(6.6);
433 Bacteria(100);Firmicutes(81.6);Clostridia(81.6);Clostridiales(81.6);Lachnospiraceae(71.4);Robinsoniella(38.4);
434 Archaea(24.9);"Euryarchaeota"(23.6);"Methanomicrobia"(10.9);Methanosarcinales(9.8);Methanosarcinaceae(9.6);Methanimicrococcus(9.4);
435 Bacteria(100);Firmicutes(96);Clostridia(96);Clostridiales(96);Ruminococcaceae(95.9);Anaerotruncus(40.5);
436 Bacteria(100);Firmicutes(82.9);Clostridia(82.9);Clostridiales(82.9);Lachnospiraceae(74.7);Dorea(63.8);
437 Bacteria(100);Firmicutes(100);Negativicutes(100);Selenomonadales(100);Veillonellaceae(100);Dialister(99.9);
438 Eukaryota(15.4);Arthropoda(7.5);Insecta(7.5);Coleoptera(7.5);Tenebrionidae(7.5);Tribolium(7.5);
439 Bacteria(100);Firmicutes(97.8);Clostridia(97.8);Clostridiales(97.8);Ruminococcaceae(97.7);Flavonifractor(37.7);
440 Bacteria(87.4);Firmicutes(34.3);Clostridia(23.1);Clostridiales(15.4);Lachnospiraceae(5.6);Cellulosilyticum(3);
441 Archaea(9.9);"Euryarchaeota"(7.8);Thermoplasmata(5.2);Thermoplasmatales(5.2);Ferroplasmaceae(5.1);Ferroplasma(5.1);
442 Bacteria(100);"Proteobacteria"(100);Betaproteobacteria(100);Neisseriales(100);Neisseriaceae(100);Neisseria(100);
443 Bacteria(100);Firmicutes(100);Clostridia(100);Clostridiales(100);Lachnospiraceae(98.9);Coprococcus(40.4);
444 Bacteria(100);"Bacteroidetes"(100);"Bacteroidia"(100);"Bacteroidales"(100);"Prevotellaceae"(100);Prevotella(100);
445 Eukaryota(10.1);Arthropoda(6.4);Insecta(6.4);Coleoptera(6.4);Tenebrionidae(6.4);Tribolium(6.4);
446 Bacteria(100);"Bacteroidetes"(77);"Bacteroidia"(73.9);"Bacteroidales"(73.9);"Porphyromonadaceae"(72.9);Tannerella(35.8);
447 Eukaryota(25.5);Chordata(19.6);Mammalia(19.6);Rodentia(17.3);Muridae(17.3);Mus(17.3);
448 Bacteria(100);Firmicutes(100);Clostridia(100);Clostridiales(100);Lachnospiraceae(98.4);Coprococcus(13.7);
449 Bacteria(100);"Bacteroidetes"(99.1);"Bacteroidia"(96.8);"Bacteroidales"(96.8);"Porphyromonadaceae"(83.3);Tannerella(49.5);
450 Eukaryota(14.5);Chordata(11.8);Mammalia(11.8);Rodentia(11.8);Muridae(11.8);Mus(11.8);
451 Bacteria(100);"Bacteroidetes"(100);"Bacteroidia"(99.8);"Bacteroidales"(99.8);"Porphyromonadaceae"(98.5);Barnesiella(79.9);
452 Eukaryota(14.6);Arthropoda(9.3);Insecta(9.3);Coleoptera(9.3);Tenebrionidae(9.3);Tribolium(9.3);
453 Bacteria(100);"Bacteroidetes"(99.8);"Bacteroidia"(98.5);"Bacteroidales"(98.5);"Porphyromonadaceae"(96.2);Barnesiella(71.8);
454 Bacteria(100);Firmicutes(73.6);Clostridia(73.1);Clostridiales(73.1);Clostridiaceae_1(69.5);Anaerobacter(68.7);
455 Bacteria(100);"Bacteroidetes"(100);"Bacteroidia"(98.7);"Bacteroidales"(98.7);"Porphyromonadaceae"(96.3);Barnesiella(73.4);
456 Bacteria(99.9);"Tenericutes"(41.2);Mollicutes(41.2);Anaeroplasmatales(40.9);Anaeroplasmataceae(40.9);Asteroleplasma(40.9);
457 Bacteria(100);Firmicutes(100);Clostridia(100);Clostridiales(100);Lachnospiraceae(77.5);Roseburia(31.4);
458 Archaea(17.3);"Euryarchaeota"(14.5);"Methanomicrobia"(7.6);Methanosarcinales(5.8);Methanosarcinaceae(5.6);Methanimicrococcus(5.6);
459 Eukaryota(6.6);Arthropoda(4.6);Insecta(4.6);Coleoptera(4.6);Tenebrionidae(4.6);Tribolium(4.6);
460 Bacteria(86.7);"Thermodesulfobacteria"(5.8);Thermodesulfobacteria(5.8);Thermodesulfobacteriales(5.8);Thermodesulfobacteriaceae(5.8);Caldimicrobium(5.8);
461 Eukaryota(26.5);Chordata(25.2);Mammalia(25.2);Rodentia(19.4);Muridae(19.4);Mus(19.4);
462 Bacteria(100);"Bacteroidetes"(99.9);"Bacteroidia"(97.5);"Bacteroidales"(97.5);"Porphyromonadaceae"(94.2);Barnesiella(71.3);
463 Bacteria(85.3);"Bacteroidetes"(37.6);"Bacteroidia"(18.1);"Bacteroidales"(18.1);"Porphyromonadaceae"(11.8);Proteiniphilum(8.8);
464 Archaea(20.3);"Euryarchaeota"(19);Thermoplasmata(7.3);Thermoplasmatales(7.3);Ferroplasmaceae(5.7);Ferroplasma(5.7);
465 Eukaryota(11.8);Streptophyta(10.3);Liliopsida(10.3);Poales(10.3);Poaceae(10.3);Zea(10.3);
466 Bacteria(99.6);"Actinobacteria"(70.3);Actinobacteria(70.3);Coriobacteriales(63.6);Coriobacteriaceae(63.6);Asaccharobacter(28.5);
467 Eukaryota(24.2);Chordata(22);Mammalia(22);Rodentia(16.6);Muridae(16.6);Mus(16.6);
468 Eukaryota(8.7);Arthropoda(4.9);Insecta(4.9);Coleoptera(4.9);Tenebrionidae(4.9);Tribolium(4.9);
469 Bacteria(100);Firmicutes(100);Clostridia(100);Clostridiales(100);Lachnospiraceae(99.1);Coprococcus(28.4);
470 Bacteria(83.6);"Proteobacteria"(36.5);Gammaproteobacteria(23.4);Chromatiales(9.9);Halothiobacillaceae(9.7);Thioalkalibacter(9.6);
471 Eukaryota(23.2);Chordata(23);Mammalia(23);Rodentia(17.1);Muridae(17.1);Mus(17.1);
472 Bacteria(100);Firmicutes(100);Clostridia(100);Clostridiales(100);Ruminococcaceae(100);Flavonifractor(62.6);
473 Bacteria(100);Firmicutes(100);Clostridia(100);Clostridiales(100);Lachnospiraceae(99.9);Clostridium_XlVa(58.6);
474 Eukaryota(15.7);Arthropoda(11.5);Insecta(11.5);Coleoptera(11.5);Tenebrionidae(11.5);Tribolium(11.5);
475 Bacteria(100);Firmicutes(97.7);Clostridia(97.2);Clostridiales(97);Ruminococcaceae(49.5);Anaerotruncus(20.7);
476 Bacteria(81.1);"Proteobacteria"(27.7);Gammaproteobacteria(12.5);"Vibrionales"(5.9);Vibrionaceae(5.9);Grimontia(5.9);
477 Bacteria(100);Firmicutes(100);Clostridia(100);Clostridiales(100);Ruminococcaceae(82.5);Ruminococcus(82.2);
478 Bacteria(86);"Bacteroidetes"(30.4);"Bacteroidia"(9.9);"Bacteroidales"(9.9);"Porphyromonadaceae"(5.7);Petrimonas(3.2);
479 Eukaryota(7.2);Streptophyta(6.1);Liliopsida(6.1);Poales(6.1);Poaceae(6.1);Zea(6.1);
480 Bacteria(90.2);"Proteobacteria"(31.4);Betaproteobacteria(9.9);Neisseriales(7.8);Neisseriaceae(7.8);Stenoxybacter(3.5);
481 Eukaryota(13.4);Streptophyta(12.4);Liliopsida(12.4);Poales(12.4);Poaceae(12.4);Zea(12.4);
482 Eukaryota(6.2);Arthropoda(3.8);Insecta(3.8);Coleoptera(3.8);Tenebrionidae(3.8);Tribolium(3.8);
483 Bacteria(78.6);Firmicutes(22.7);Clostridia(17.8);Clostridiales(15.6);Clostridiales_Incertae_Sedis_XI(7.1);Parvimonas(6.4);
484 Bacteria(100);Firmicutes(97.6);Clostridia(95.2);Clostridiales(94.9);Lachnospiraceae(62.1);Marvinbryantia(21.9);
485 Eukaryota(14.5);Streptophyta(12.2);Liliopsida(12.2);Poales(12.2);Poaceae(12.2);Zea(12.2);
486 Bacteria(100);Firmicutes(97.5);Clostridia(97.5);Clostridiales(97.5);Lachnospiraceae(97.3);Robinsoniella(78.5);
487 Eukaryota(13.5);Chordata(7.1);Mammalia(7.1);Primates(2.3);Hominidae(2.3);Homo(2.3);
488 Bacteria(100);Firmicutes(98.7);Clostridia(98.7);Clostridiales(98.7);Lachnospiraceae(90.7);Syntrophococcus(27.3);
489 Archaea(17);"Euryarchaeota"(10.8);Thermoplasmata(8);Thermoplasmatales(8);Thermoplasmatales_incertae_sedis(7.4);Thermogymnomonas(7.4);
490 Bacteria(81);"Lentisphaerae"(7.3);"Lentisphaeria"(7.3);Lentisphaerales(7.3);"Lentisphaeraceae"(7.3);Lentisphaera(7.3);
491 Eukaryota(16.5);Streptophyta(14.6);Liliopsida(14.6);Poales(14.6);Poaceae(14.6);Zea(14.6);
492 Bacteria(100);"Bacteroidetes"(100);"Bacteroidia"(98.6);"Bacteroidales"(98.6);"Porphyromonadaceae"(97.6);Barnesiella(64.3);
493 Bacteria(100);"Bacteroidetes"(100);"Bacteroidia"(100);"Bacteroidales"(100);Bacteroidaceae(100);Bacteroides(100);
494 Bacteria(100);Firmicutes(100);Clostridia(100);Clostridiales(100);Ruminococcaceae(100);Oscillibacter(94);
495 Bacteria(100);Firmicutes(90.6);Erysipelotrichia(79.5);Erysipelotrichales(79.5);Erysipelotrichaceae(79.5);Sharpea(20.3);
496 Archaea(19.8);"Euryarchaeota"(17);"Methanomicrobia"(9.9);Methanosarcinales(7.7);Methanosarcinaceae(7.7);Methanohalobium(4.5);
497 Eukaryota(13.3);Streptophyta(10.6);Liliopsida(10.6);Poales(10.6);Poaceae(10.6);Zea(10.6);
498 Eukaryota(22.3);Chordata(12.5);Mammalia(12.5);Primates(3.1);Hominidae(3.1);Homo(3.1);
499 Bacteria(85.2);"Proteobacteria"(37.6);Gammaproteobacteria(16.5);Legionellales(5.5);Coxiellaceae(5.5);Coxiella(5.5);
500 Eukaryota(11.3);Streptophyta(9.8);Liliopsida(9.8);Poales(9.8);Poaceae(9.8);Zea(9.8);
501 Eukaryota(19.6);Streptophyta(18);Liliopsida(18);Poales(18);Poaceae(18);Zea(18);
502 Eukaryota(13.7);Chordata(9.9);Mammalia(9.9);Rodentia(9.9);Muridae(9.9);Mus(9.9);
503 Bacteria(82.1);"Bacteroidetes"(30);"Sphingobacteria"(16.4);"Sphingobacteriales"(16.4);"Cyclobacteriaceae"(8.4);Fontibacter(7.2);
504 Eukaryota(6);Arthropoda(4);Insecta(4);Coleoptera(4);Tenebrionidae(4);Tribolium(4);
505 Bacteria(84.3);"Bacteroidetes"(21.6);"Sphingobacteria"(14.9);"Sphingobacteriales"(14.9);Cytophagaceae(9.9);Leadbetterella(4.8);
506 Bacteria(100);Firmicutes(99.9);Clostridia(69.9);Clostridiales(69.9);Lachnospiraceae(67.6);Syntrophococcus(28.2);
507 Bacteria(91.2);"Proteobacteria"(27.7);Alphaproteobacteria(13.1);Rhizobiales(4.4);Bradyrhizobiaceae(2.7);Agromonas(2.7);
508 Bacteria(88);"Bacteroidetes"(26.1);"Sphingobacteria"(15.8);"Sphingobacteriales"(15.8);Sphingobacteriaceae(4.3);Pseudosphingobacterium(3.2);
509 Bacteria(100);Firmicutes(100);Clostridia(100);Clostridiales(100);Lachnospiraceae(53.4);Robinsoniella(23.2);
510 Eukaryota(26.6);Chordata(16.2);Mammalia(16.2);Rodentia(13.4);Muridae(13.4);Mus(13.4);
511 Bacteria(100);Firmicutes(100);Clostridia(100);Clostridiales(100);Ruminococcaceae(100);Oscillibacter(75);
512 Bacteria(85.2);"Proteobacteria"(40.9);Gammaproteobacteria(25.3);Chromatiales(13.2);Halothiobacillaceae(11.8);Thioalkalibacter(11.7);
513 Bacteria(100);Firmicutes(100);Clostridia(100);Clostridiales(100);Ruminococcaceae(100);Pseudoflavonifractor(74.5);

514 Bacteria(92.8);"Proteobacteria"(31.1);Alphaproteobacteria(13.3);Rhizobiales(5.5);Bradyrhizobiaceae(3.2);Agromonas(3.1);
515 Bacteria(100);Firmicutes(100);Clostridia(100);Clostridiales(100);Lachnospiraceae(93.2);Robinsoniella(29.5);
516 Bacteria(89.9);Firmicutes(31.4);Clostridia(25.8);Clostridiales(23.4);Clostridiales_Incertae_Sedis_XI(5.8);Parvimonas(4.6);
517 Bacteria(100);Firmicutes(99.7);Clostridia(99.7);Clostridiales(99.7);Ruminococcaceae(69.6);Oscillibacter(60.7);
518 Bacteria(100);Firmicutes(96.2);Clostridia(96);Clostridiales(96);Lachnospiraceae(80.8);Robinsoniella(30.8);
519 Bacteria(100);Firmicutes(61.6);Clostridia(61.5);Clostridiales(61.5);Lachnospiraceae(60.1);Shuttleworthia(30.2);
520 Bacteria(100);Firmicutes(56.2);Clostridia(48.6);Clostridiales(47.6);Ruminococcaceae(24);Acetivibrio(12.7);
521 Eukaryota(12);Streptophyta(7.4);Liliopsida(7.4);Poales(7.4);Poaceae(7.4);Zea(7.4);
522 Eukaryota(23.9);Chordata(16.1);Mammalia(16.1);Rodentia(16.1);Muridae(16.1);Mus(16.1);
523 Eukaryota(20.3);Chordata(18.9);Mammalia(18.9);Rodentia(15.8);Muridae(15.8);Mus(15.8);
524 Bacteria(87.7);"Proteobacteria"(28.5);Deltaproteobacteria(7.9);Desulfobacterales(3.5);Desulfobacteraceae(2);Desulfatiferula(1.8);
525 Bacteria(100);"Bacteroidetes"(100);"Bacteroidia"(100);"Bacteroidales"(100);"Prevotellaceae"(99.9);Prevotella(82.1);
526 Bacteria(100);"Tenericutes"(98);Mollicutes(98);Anaeroplasmatales(65.5);Anaeroplasmataceae(65.5);Anaeroplasma(65.5);
527 Eukaryota(9.7);Arthropoda(6.3);Insecta(6.3);Coleoptera(6.3);Tenebrionidae(6.3);Tribolium(6.3);
528 Eukaryota(27.2);Chordata(24.5);Mammalia(24.5);Rodentia(18.6);Muridae(18.6);Mus(18.6);
529 Bacteria(100);Firmicutes(100);Clostridia(94.5);Clostridiales(94.5);Lachnospiraceae(90.6);Coprococcus(46.1);
530 Bacteria(100);Firmicutes(98.3);Erysipelotrichia(98.3);Erysipelotrichales(98.3);Erysipelotrichaceae(98.3);Clostridium_XVIII(94.2);
531 Bacteria(100);Firmicutes(60.7);Clostridia(60.7);Clostridiales(60.7);Lachnospiraceae(55);Marvinbryantia(12.2);
532 Bacteria(100);Firmicutes(100);Clostridia(100);Clostridiales(100);Lachnospiraceae(91.5);Clostridium_XlVa(31.2);
533 Bacteria(100);Firmicutes(99.7);Clostridia(99.7);Clostridiales(99.7);Ruminococcaceae(99.7);Oscillibacter(87.5);
534 Bacteria(88.4);"Bacteroidetes"(31.6);"Bacteroidia"(15.3);"Bacteroidales"(15.3);"Porphyromonadaceae"(8.4);Proteiniphilum(4.2);
535 Bacteria(100);"Bacteroidetes"(99.1);"Bacteroidia"(95.4);"Bacteroidales"(95.4);"Porphyromonadaceae"(83.4);Tannerella(38.5);
536 Bacteria(100);Firmicutes(99.9);Clostridia(99.9);Clostridiales(99.9);Ruminococcaceae(99.7);Clostridium_IV(63.1);
537 Eukaryota(6.1);Arthropoda(4.2);Insecta(4.2);Coleoptera(4.2);Tenebrionidae(4.2);Tribolium(4.2);
538 Eukaryota(15.4);Arthropoda(8.5);Insecta(8.5);Coleoptera(8.5);Tenebrionidae(8.5);Tribolium(8.5);
539 Bacteria(100);Firmicutes(99.8);Clostridia(99.8);Clostridiales(99.8);Ruminococcaceae(85.9);Anaerotruncus(35.1);
540 Bacteria(100);Firmicutes(99.7);Clostridia(99.6);Clostridiales(99.6);Lachnospiraceae(86.8);Moryella(23.6);
541 Bacteria(100);Firmicutes(86.7);Clostridia(86.7);Clostridiales(86.7);Lachnospiraceae(86.7);Syntrophococcus(30.2);
542 Bacteria(100);Firmicutes(85.6);Clostridia(85.5);Clostridiales(85.5);Lachnospiraceae(78.1);Marvinbryantia(32.2);
543 Bacteria(100);Firmicutes(93.9);Clostridia(84.8);Clostridiales(84.7);Peptococcaceae_1(48.6);Dehalobacter(45.9);
544 Archaea(16.4);"Euryarchaeota"(14.4);Halobacteria(7.4);Halobacteriales(7.4);Halobacteriaceae(7.4);Halonotius(3);
545 Bacteria(92.3);"Proteobacteria"(33.5);Alphaproteobacteria(14.5);Rhizobiales(5);Bradyrhizobiaceae(2.7);Agromonas(2.7);
546 Eukaryota(14.4);Chordata(11.5);Mammalia(11.5);Rodentia(11.5);Muridae(11.5);Mus(11.5);
547 Bacteria(100);Firmicutes(100);Clostridia(100);Clostridiales(100);Lachnospiraceae(99.6);Pseudobutyrivibrio(40.2);
548 Bacteria(100);Firmicutes(100);Clostridia(99.9);Clostridiales(99.9);Ruminococcaceae(76.6);Clostridium_IV(51.1);
549 Bacteria(100);Firmicutes(99.9);Clostridia(99.9);Clostridiales(99.9);Ruminococcaceae(99.8);Oscillibacter(86.4);
550 Bacteria(100);Firmicutes(99.8);Clostridia(99.8);Clostridiales(99.6);Ruminococcaceae(92.9);Oscillibacter(76.1);
551 Bacteria(100);"Bacteroidetes"(49.1);"Bacteroidia"(44.8);"Bacteroidales"(44.8);"Porphyromonadaceae"(44.1);Barnesiella(42.3);
552 Bacteria(86.9);"Actinobacteria"(9.7);Actinobacteria(9.7);Actinomycetales(8.6);Micromonosporaceae(6.9);Catelliglobosispora(6.5);
553 Bacteria(100);"Bacteroidetes"(100);"Bacteroidia"(100);"Bacteroidales"(100);"Rikenellaceae"(100);Alistipes(100);
554 Bacteria(100);Firmicutes(53.5);Clostridia(53.5);Clostridiales(53.5);Ruminococcaceae(53.5);Clostridium_IV(34.6);
555 Eukaryota(26.8);Chordata(23.9);Mammalia(23.9);Rodentia(17.9);Muridae(17.9);Mus(17.9);
556 Bacteria(90.3);"Proteobacteria"(31.9);Alphaproteobacteria(13.4);Rhizobiales(7.2);Bradyrhizobiaceae(4.8);Agromonas(4.8);
557 Bacteria(100);Firmicutes(88.3);Clostridia(83.1);Clostridiales(82.6);Clostridiales_Incertae_Sedis_XII(41.2);Acidaminobacter(32.9);
558 Bacteria(100);Firmicutes(100);Clostridia(100);Clostridiales(100);Ruminococcaceae(100);Acetanaerobacterium(82.7);
559 Eukaryota(8);Streptophyta(5.9);Liliopsida(5.9);Poales(5.9);Poaceae(5.9);Zea(5.9);
560 Bacteria(85.6);"Bacteroidetes"(32.4);"Bacteroidia"(15);"Bacteroidales"(15);"Porphyromonadaceae"(6.3);Petrimonas(4.4);
561 Bacteria(100);"Bacteroidetes"(100);"Bacteroidia"(97.9);"Bacteroidales"(97.9);"Porphyromonadaceae"(97);Barnesiella(73.1);
562 Bacteria(100);"Bacteroidetes"(96);"Bacteroidia"(87.9);"Bacteroidales"(87.9);"Porphyromonadaceae"(87.1);Barnesiella(64.3);
563 Eukaryota(15.4);Streptophyta(5.7);Liliopsida(5.7);Poales(5.7);Poaceae(5.7);Zea(5.7);
564 Bacteria(100);Firmicutes(100);Clostridia(100);Clostridiales(100);Lachnospiraceae(95.8);Lachnospiracea_incertae_sedis(52.6);
565 Bacteria(100);Firmicutes(98.6);Clostridia(98.5);Clostridiales(98.4);Ruminococcaceae(85.7);Anaerotruncus(34.9);
566 Bacteria(81.7);"Proteobacteria"(40.6);Gammaproteobacteria(23.7);Chromatiales(11.2);Halothiobacillaceae(11.2);Thioalkalibacter(11);
567 Bacteria(100);"Bacteroidetes"(75.3);"Bacteroidia"(73.3);"Bacteroidales"(73.3);"Porphyromonadaceae"(67.8);Tannerella(35.1);
568 Eukaryota(27.7);Arthropoda(14.7);Insecta(14.7);Coleoptera(14.7);Tenebrionidae(14.7);Tribolium(14.7);
569 Bacteria(100);Firmicutes(99.9);Clostridia(99.9);Clostridiales(99.9);Ruminococcaceae(95.4);Papillibacter(28.6);
570 Bacteria(100);Firmicutes(98.8);Clostridia(98.8);Clostridiales(98.8);Ruminococcaceae(98.7);Flavonifractor(84.9);
571 Bacteria(100);Firmicutes(96);Clostridia(58.3);Clostridiales(58.2);Ruminococcaceae(49.6);Oscillibacter(33.8);
572 Bacteria(100);Firmicutes(100);Clostridia(98.2);Clostridiales(98.2);Lachnospiraceae(97);Coprococcus(38.2);
573 Eukaryota(8.2);Streptophyta(6.2);Liliopsida(6.2);Poales(6.2);Poaceae(6.2);Zea(6.2);
574 Bacteria(90.3);"Proteobacteria"(37.2);Alphaproteobacteria(7.5);Rickettsiales(4.5);Anaplasmataceae(4.5);Wolbachia(4.4);
575 Bacteria(100);Firmicutes(96.9);Clostridia(83.9);Clostridiales(83);Ruminococcaceae(48.4);Pseudoflavonifractor(14.1);
576 Bacteria(100);Firmicutes(100);Clostridia(100);Clostridiales(100);Ruminococcaceae(98.8);Oscillibacter(87.6);
577 Bacteria(100);Firmicutes(100);Clostridia(100);Clostridiales(100);Ruminococcaceae(98);Ruminococcus(52.1);
578 Bacteria(100);"Bacteroidetes"(64.3);"Bacteroidia"(58);"Bacteroidales"(58);"Porphyromonadaceae"(51);Tannerella(29.1);
579 Eukaryota(22.2);Streptophyta(19.7);Liliopsida(19.7);Poales(19.7);Poaceae(19.7);Zea(19.7);
580 Bacteria(100);Firmicutes(99.8);Clostridia(99.8);Clostridiales(99.8);Lachnospiraceae(97.5);Marvinbryantia(38.5);
581 Bacteria(100);Firmicutes(99.9);Clostridia(99.9);Clostridiales(99.9);Lachnospiraceae(99.9);Roseburia(60.8);
582 Bacteria(100);Firmicutes(100);Clostridia(100);Clostridiales(100);Clostridiaceae_1(100);Clostridium_sensu_stricto(87.9);
583 Eukaryota(11.1);Streptophyta(8.9);Liliopsida(8.9);Poales(8.9);Poaceae(8.9);Zea(8.9);
584 Bacteria(100);Firmicutes(100);Clostridia(100);Clostridiales(100);Lachnospiraceae(74.8);Lachnospira(19.5);
585 Bacteria(82.2);"Proteobacteria"(25.2);Deltaproteobacteria(11.3);Myxococcales(8.1);Nannocystaceae(7.7);Enhygromyxa(7.3);
586 Bacteria(100);Firmicutes(82);Erysipelotrichia(79.3);Erysipelotrichales(79.3);Erysipelotrichaceae(79.3);Holdemania(61.3);
587 Bacteria(100);Firmicutes(100);Clostridia(100);Clostridiales(100);Lachnospiraceae(99.4);Clostridium_XlVa(40.1);
588 Bacteria(100);Firmicutes(95.3);Erysipelotrichia(95.3);Erysipelotrichales(95.3);Erysipelotrichaceae(95.3);Holdemania(65);
589 Bacteria(100);Firmicutes(99.9);Clostridia(99.8);Clostridiales(99.8);Clostridiaceae_1(99.8);Clostridium_sensu_stricto(84);
590 Bacteria(87.7);"Bacteroidetes"(32.7);"Bacteroidia"(12.9);"Bacteroidales"(12.9);"Porphyromonadaceae"(6.4);Proteiniphilum(3.1);
591 Bacteria(100);Firmicutes(99.9);Clostridia(99.9);Clostridiales(99.9);Lachnospiraceae(85.4);Coprococcus(33.5);
592 Bacteria(100);"Bacteroidetes"(99.9);"Bacteroidia"(99.2);"Bacteroidales"(99.2);"Porphyromonadaceae"(94.8);Tannerella(28.9);
593 Eukaryota(9.5);Arthropoda(5.3);Insecta(5.3);Coleoptera(5.3);Tenebrionidae(5.3);Tribolium(5.3);
594 Eukaryota(10.2);Streptophyta(6.3);Liliopsida(6.3);Poales(6.3);Poaceae(6.3);Zea(6.3);
595 Bacteria(100);Firmicutes(86);Clostridia(85.8);Clostridiales(85.8);Ruminococcaceae(81.9);Oscillibacter(41.1);
596 Bacteria(99.9);"Bacteroidetes"(64.2);"Bacteroidia"(46.2);"Bacteroidales"(46.2);"Porphyromonadaceae"(42.9);Barnesiella(35.8);
597 Bacteria(84.8);"Actinobacteria"(9.1);Actinobacteria(9.1);Actinomycetales(8.4);Micromonosporaceae(6.9);Catelliglobosispora(6.5);
598 Bacteria(100);Firmicutes(100);Clostridia(100);Clostridiales(100);Clostridiaceae_1(100);Clostridium_sensu_stricto(81.9);
599 Bacteria(100);Firmicutes(90.7);Clostridia(90.7);Clostridiales(90.7);Ruminococcaceae(90.7);Papillibacter(36.6);

600 Bacteria(100);Firmicutes(100);Clostridia(100);Clostridiales(100);Lachnospiraceae(98.7);Coprococcus(19.5);
601 Eukaryota(10.8);Streptophyta(9.2);Liliopsida(9.2);Poales(9.2);Poaceae(9.2);Zea(9.2);
602 Bacteria(87.6);"Bacteroidetes"(28.8);"Bacteroidia"(11.8);"Bacteroidales"(11.8);"Porphyromonadaceae"(4.8);Petrimonas(3.2);
603 Bacteria(84.1);"Bacteroidetes"(26.2);"Sphingobacteria"(11.1);"Sphingobacteriales"(11.1);"Flammeovirgaceae"(6.4);Flexithrix(5.5);
604 Bacteria(100);Firmicutes(62.5);Clostridia(61.6);Clostridiales(61.2);Ruminococcaceae(50.4);Flavonifractor(18.9);
605 Bacteria(100);Firmicutes(100);Clostridia(100);Clostridiales(100);Ruminococcaceae(100);Anaerotruncus(99.8);
606 Bacteria(100);Firmicutes(99.8);Clostridia(85.5);Clostridiales(85.5);Lachnospiraceae(84.5);Johnsonella(51.7);
607 Bacteria(87.6);"Bacteroidetes"(32.7);"Bacteroidia"(11.8);"Bacteroidales"(11.8);"Porphyromonadaceae"(7.5);Petrimonas(4.4);
608 Eukaryota(9);Streptophyta(8.5);Liliopsida(8.5);Poales(8.5);Poaceae(8.5);Zea(8.5);
609 Bacteria(100);Firmicutes(98.5);Clostridia(94.2);Clostridiales(94.2);Lachnospiraceae(81.4);Acetitomaculum(19.8);
610 Eukaryota(21.8);Chordata(21.3);Mammalia(21.3);Rodentia(16);Muridae(16);Mus(16);
611 Bacteria(86.5);"Bacteroidetes"(31.7);"Bacteroidia"(13.1);"Bacteroidales"(13.1);"Porphyromonadaceae"(5.4);Petrimonas(3.5);
612 Bacteria(86.4);"Proteobacteria"(39.5);Alphaproteobacteria(12.3);Rhodobacterales(8.9);Rhodobacteraceae(8.9);Nereida(3.5);
613 Bacteria(100);"Bacteroidetes"(58.2);"Bacteroidia"(48.5);"Bacteroidales"(48.5);"Porphyromonadaceae"(42.7);Tannerella(27.5);
614 Archaea(17.7);"Euryarchaeota"(13.6);Thermoplasmata(5.5);Thermoplasmatales(5.5);Thermoplasmatales_incertae_sedis(3.3);Thermogymnomonas(3.3);
615 Bacteria(100);"Bacteroidetes"(61);"Bacteroidia"(55.2);"Bacteroidales"(55.2);"Porphyromonadaceae"(45.8);Tannerella(34.9);
616 Bacteria(83.9);"Bacteroidetes"(27.3);Flavobacteria(19.9);"Flavobacteriales"(19.9);Flavobacteriaceae(19.4);Joostella(8.5);
617 Archaea(8.9);"Euryarchaeota"(7.7);Thermoplasmata(5.3);Thermoplasmatales(5.3);Ferroplasmaceae(5.2);Ferroplasma(5.2);
618 Bacteria(100);Firmicutes(87.6);Clostridia(85.2);Clostridiales(85.1);Ruminococcaceae(80.3);Anaerotruncus(27.4);
619 Eukaryota(16.7);Streptophyta(9.5);Liliopsida(9.5);Poales(9.5);Poaceae(9.5);Zea(9.5);
620 Eukaryota(8.2);Arthropoda(7.3);Insecta(7.3);Coleoptera(7.3);Tenebrionidae(7.3);Tribolium(7.3);
621 Eukaryota(14.4);Chordata(10.1);Mammalia(10.1);Rodentia(10.1);Muridae(10.1);Mus(10.1);
622 Eukaryota(11.9);Arthropoda(8);Insecta(8);Coleoptera(8);Tenebrionidae(8);Tribolium(8);
623 Eukaryota(12.7);Arthropoda(8.2);Insecta(8.2);Coleoptera(8.2);Tenebrionidae(8.2);Tribolium(8.2);
624 Bacteria(88);"Bacteroidetes"(31.6);"Bacteroidia"(11);"Bacteroidales"(11);"Porphyromonadaceae"(4.6);Petrimonas(2.2);
625 Archaea(26.4);"Euryarchaeota"(25.6);"Methanomicrobia"(12.4);Methanomicrobiales(12.4);Methanomicrobiaceae(7.8);Methanosphaerula(5.8);
626 Archaea(24.4);"Euryarchaeota"(19.8);Halobacteria(14);Halobacteriales(14);Halobacteriaceae(14);Halonotius(6.6);
627 Eukaryota(11.9);Streptophyta(7.1);Liliopsida(7.1);Poales(7.1);Poaceae(7.1);Zea(7.1);
628 Bacteria(88.2);Firmicutes(28.4);Clostridia(21.6);Clostridiales(19.1);Clostridiales_Incertae_Sedis_XI(4.3);Parvimonas(3);
629 Eukaryota(10.4);Chordata(6.2);Mammalia(6.2);Rodentia(6.2);Muridae(6.2);Mus(6.2);
630 Bacteria(100);Firmicutes(99.6);Clostridia(99.6);Clostridiales(99.3);Ruminococcaceae(71.4);Flavonifractor(30.4);
631 Eukaryota(21.2);Chordata(10.9);Mammalia(10.9);Rodentia(8.2);Muridae(8.2);Mus(8.2);
632 Eukaryota(8.3);Arthropoda(6.4);Insecta(6.4);Coleoptera(6.4);Tenebrionidae(6.4);Tribolium(6.4);
633 Bacteria(100);Firmicutes(94.1);Clostridia(94.1);Clostridiales(94.1);Lachnospiraceae(85.7);Parasporobacterium(52.2);
634 Bacteria(100);"Bacteroidetes"(58.5);"Bacteroidia"(43.3);"Bacteroidales"(43.3);"Porphyromonadaceae"(35.8);Petrimonas(15.2);
635 Bacteria(100);Firmicutes(100);Clostridia(72.1);Clostridiales(72.1);Ruminococcaceae(71.7);Acetanaerobacterium(17.7);
636 Bacteria(100);Firmicutes(100);Clostridia(100);Clostridiales(100);Ruminococcaceae(99.8);Oscillibacter(63.3);
637 Bacteria(100);Firmicutes(83.1);Clostridia(83.1);Clostridiales(83.1);Ruminococcaceae(81.7);Clostridium_IV(39.9);
638 Eukaryota(26.4);Chordata(23.2);Mammalia(23.2);Rodentia(19.8);Muridae(19.8);Mus(19.8);
639 Eukaryota(10.9);Streptophyta(9.6);Liliopsida(9.6);Poales(9.6);Poaceae(9.6);Zea(9.6);
640 Bacteria(100);"Bacteroidetes"(41.2);"Bacteroidia"(40.7);"Bacteroidales"(40.7);Bacteroidaceae(40.7);Anaerorhabdus(40.7);
641 Bacteria(85.7);Firmicutes(25.5);Clostridia(22.2);Clostridiales(20.8);Clostridiales_Incertae_Sedis_XI(7);Parvimonas(5.7);
642 Eukaryota(12.6);Chordata(7.7);Mammalia(7.7);Primates(2.9);Hominidae(2.9);Homo(2.9);
643 Bacteria(100);Firmicutes(85.8);Clostridia(80.7);Clostridiales(80.7);Ruminococcaceae(78.3);Acetivibrio(49.3);
644 Archaea(18.6);"Euryarchaeota"(15.3);"Methanomicrobia"(7.6);Methanosarcinales(5.3);Methanosarcinaceae(5.2);Methanohalobium(3.4);
645 Bacteria(100);Firmicutes(97);Clostridia(96.5);Clostridiales(96.5);Ruminococcaceae(52.5);Ethanoligenens(17);
646 Eukaryota(12.9);Streptophyta(11.7);Liliopsida(11.7);Poales(11.7);Poaceae(11.7);Zea(11.7);
647 Eukaryota(11.7);Arthropoda(8.1);Insecta(8.1);Coleoptera(8.1);Tenebrionidae(8.1);Tribolium(8.1);
648 Bacteria(86.3);"Bacteroidetes"(31.6);"Bacteroidia"(11.8);"Bacteroidales"(11.8);"Porphyromonadaceae"(7.4);Petrimonas(5.6);
649 Eukaryota(22.2);Chordata(8.7);Mammalia(8.7);Primates(1.5);Hominidae(1.5);Homo(1.5);
650 Eukaryota(15.8);Arthropoda(9.3);Insecta(9.3);Coleoptera(9.3);Tenebrionidae(9.3);Tribolium(9.3);
651 Eukaryota(11.7);Streptophyta(10.4);Liliopsida(10.4);Poales(10.4);Poaceae(10.4);Zea(10.4);
652 Bacteria(100);Firmicutes(100);Clostridia(100);Clostridiales(100);Lachnospiraceae(100);Lachnospiracea_incertae_sedis(60.6);
653 Eukaryota(10.3);Arthropoda(5.2);Insecta(5.2);Coleoptera(5.2);Tenebrionidae(5.2);Tribolium(5.2);
654 Bacteria(85.7);"Bacteroidetes"(22.7);"Sphingobacteria"(12.6);"Sphingobacteriales"(12.6);Sphingobacteriaceae(3.4);Pseudosphingobacterium(2.2);
655 Bacteria(100);Firmicutes(100);Clostridia(100);Clostridiales(100);Ruminococcaceae(85.4);Oscillibacter(85.3);
656 Bacteria(100);Firmicutes(99.3);Clostridia(99.3);Clostridiales(99.3);Lachnospiraceae(90.2);Anaerostipes(38.9);
657 Eukaryota(43.6);Chordata(39.8);Mammalia(39.8);Rodentia(30.8);Muridae(30.8);Mus(30.8);
658 Bacteria(100);Firmicutes(100);Clostridia(100);Clostridiales(100);Lachnospiraceae(100);Hespellia(38.1);
659 Bacteria(84.9);"Elusimicrobia"(4.8);Elusimicrobia(4.8);Elusimicrobiales(4.8);Elusimicrobiaceae(4.8);Elusimicrobium(4.8);
660 Archaea(25);"Euryarchaeota"(22.3);"Methanomicrobia"(16.5);Methanosarcinales(12.3);Methanosarcinaceae(12.3);Methanohalobium(9.1);
661 Bacteria(100);Firmicutes(99.9);Clostridia(99.9);Clostridiales(99.9);Ruminococcaceae(71.8);Anaerotruncus(66.6);
662 Eukaryota(18.9);Streptophyta(9.8);Liliopsida(9.8);Poales(9.8);Poaceae(9.8);Zea(9.8);
663 Bacteria(87.2);"Bacteroidetes"(35.7);"Bacteroidia"(16);"Bacteroidales"(16);"Porphyromonadaceae"(8.7);Petrimonas(4.9);
664 Eukaryota(12.4);Streptophyta(9.5);Liliopsida(9.5);Poales(9.5);Poaceae(9.5);Zea(9.5);
665 Eukaryota(12.5);Streptophyta(9.4);Liliopsida(9.4);Poales(9.4);Poaceae(9.4);Zea(9.4);
666 Eukaryota(17);Chordata(12.9);Mammalia(12.9);Rodentia(12.9);Muridae(12.9);Mus(12.9);
667 Eukaryota(7.4);Arthropoda(3.6);Insecta(3.6);Coleoptera(3.6);Tenebrionidae(3.6);Tribolium(3.6);
668 Bacteria(100);Firmicutes(99.1);Clostridia(99.1);Clostridiales(99.1);Lachnospiraceae(95.1);Marvinbryantia(16);
669 Bacteria(100);Firmicutes(93.3);Clostridia(93.3);Clostridiales(93.3);Lachnospiraceae(90.6);Lactonifactor(40);
670 Eukaryota(9.7);Streptophyta(8.1);Liliopsida(8.1);Poales(8.1);Poaceae(8.1);Zea(8.1);
671 Eukaryota(15);Streptophyta(13.2);Liliopsida(13.2);Poales(13.2);Poaceae(13.2);Zea(13.2);
672 Bacteria(100);Firmicutes(99.6);Clostridia(99.6);Clostridiales(99.6);Lachnospiraceae(98.6);Lactonifactor(16.2);
673 Bacteria(100);Firmicutes(98);Erysipelotrichia(92.5);Erysipelotrichales(92.5);Erysipelotrichaceae(92.5);Coprobacillus(63.9);
674 Eukaryota(11.8);Arthropoda(6.7);Insecta(6.7);Coleoptera(6.7);Tenebrionidae(6.7);Tribolium(6.7);
675 Bacteria(100);Firmicutes(98.4);Clostridia(96.3);Clostridiales(96.3);Ruminococcaceae(86.7);Ethanoligenens(26.9);
676 Eukaryota(9.8);Streptophyta(8.3);Liliopsida(8.3);Poales(8.3);Poaceae(8.3);Zea(8.3);
677 Bacteria(99.9);Firmicutes(65.8);Clostridia(60.9);Clostridiales(60.6);Clostridiales_Incertae_Sedis_XII(24);Fusibacter(17.9);
678 Eukaryota(11.6);Streptophyta(4.2);Liliopsida(4.2);Poales(4.2);Poaceae(4.2);Zea(4.2);
679 Bacteria(100);Firmicutes(99.8);Clostridia(99.8);Clostridiales(99.8);Ruminococcaceae(68.8);Sporobacter(39.2);
680 Eukaryota(12.4);Chordata(9.3);Mammalia(9.3);Rodentia(9.3);Muridae(9.3);Mus(9.3);
681 Bacteria(100);Firmicutes(100);Clostridia(100);Clostridiales(100);Lachnospiraceae(99.6);Lachnospiracea_incertae_sedis(67.1);
682 Eukaryota(26.4);Chordata(25.8);Mammalia(25.8);Rodentia(19.2);Muridae(19.2);Mus(19.2);
683 Bacteria(86);"Elusimicrobia"(3.2);Elusimicrobia(3.2);Elusimicrobiales(3.2);Elusimicrobiaceae(3.2);Elusimicrobium(3.2);
684 Eukaryota(10.5);Chordata(8.5);Mammalia(8.5);Rodentia(7.6);Muridae(7.6);Mus(7.6);
685 Eukaryota(12.7);Chordata(9.2);Mammalia(9.2);Rodentia(9.2);Muridae(9.2);Mus(9.2);

686 Bacteria(100);"Bacteroidetes"(100);"Bacteroidia"(100);"Bacteroidales"(100);"Prevotellaceae"(100);Prevotella(99.8);
687 Eukaryota(6.3);Arthropoda(3.7);Insecta(3.7);Coleoptera(3.7);Tenebrionidae(3.7);Tribolium(3.7);
688 Archaea(19.4);"Euryarchaeota"(16.4);"Methanomicrobia"(9.6);Methanosarcinales(6.3);Methanosarcinaceae(6.2);Methanohalobium(3.3);
689 Eukaryota(13.9);Streptophyta(5.1);Liliopsida(5.1);Poales(5.1);Poaceae(5.1);Zea(5.1);
690 Bacteria(100);Firmicutes(90.2);Clostridia(90.2);Clostridiales(90.2);Peptostreptococcaceae(84.8);Sporacetigenium(66.1);
691 Archaea(23.5);"Euryarchaeota"(18.2);"Methanomicrobia"(14.1);Methanosarcinales(9.3);Methanosarcinaceae(8.7);Methanohalobium(6.5);
692 Bacteria(100);Firmicutes(94.1);Clostridia(94);Clostridiales(94);Lachnospiraceae(92.7);Marvinbryantia(35.1);
693 Bacteria(86.5);Cyanobacteria_Chloroplast(5.9);Chloroplast(5.9);Chloroplast_order_incertae_sedis(5.9);Chloroplast(5.9);Chlorarachniophyceae(5.9);
694 Eukaryota(13.8);Streptophyta(11.4);Liliopsida(11.4);Poales(11.4);Poaceae(11.4);Zea(11.4);
695 Eukaryota(9.2);Streptophyta(8.4);Liliopsida(8.4);Poales(8.4);Poaceae(8.4);Zea(8.4);
696 Bacteria(100);"Actinobacteria"(100);Actinobacteria(100);Actinomycetales(100);Corynebacteriaceae(99.9);Corynebacterium(99.8);
697 Eukaryota(15);Streptophyta(11.8);Liliopsida(11.8);Poales(11.8);Poaceae(11.8);Zea(11.8);
698 Bacteria(100);Firmicutes(100);Clostridia(100);Clostridiales(100);Lachnospiraceae(100);Johnsonella(65.6);
699 Eukaryota(24);Streptophyta(16.3);Liliopsida(16.3);Poales(16.3);Poaceae(16.3);Zea(16.3);
700 Bacteria(100);"Bacteroidetes"(100);"Bacteroidia"(100);"Bacteroidales"(100);"Porphyromonadaceae"(100);Parabacteroides(91.3);
701 Bacteria(100);Firmicutes(96.3);Clostridia(96.3);Clostridiales(96.3);Peptostreptococcaceae(87.9);Sporacetigenium(53.4);
702 Archaea(16.4);"Euryarchaeota"(15.9);Thermoplasmata(8.4);Thermoplasmatales(8.4);Thermoplasmatales_incertae_sedis(6);Thermogymnomonas(6);
703 Bacteria(100);Firmicutes(69.2);Clostridia(61.6);Clostridiales(61.6);Lachnospiraceae(55.6);Oribacterium(21.6);
704 Bacteria(100);Firmicutes(99.9);Clostridia(98.1);Clostridiales(98);Lachnospiraceae(78.8);Clostridium_XlVb(55.2);
705 Eukaryota(16.8);Chordata(13.2);Mammalia(13.2);Rodentia(13.2);Muridae(13.2);Mus(13.2);
706 Eukaryota(11.6);Streptophyta(11.1);Liliopsida(11.1);Poales(11.1);Poaceae(11.1);Zea(11.1);
707 Eukaryota(10.9);Streptophyta(9.3);Liliopsida(9.3);Poales(9.3);Poaceae(9.3);Zea(9.3);
708 Bacteria(80.3);Firmicutes(25.6);Clostridia(19.2);Clostridiales(14.3);Peptococcaceae_2(5.2);Cryptanaerobacter(5.1);
709 Eukaryota(18.8);Arthropoda(12.2);Insecta(12.2);Coleoptera(12.2);Tenebrionidae(12.2);Tribolium(12.2);
710 Bacteria(89);Firmicutes(26.1);Clostridia(18.7);Clostridiales(15.5);Incertae_Sedis_XI(3.6);Anaerosphaera(3.6);
711 Bacteria(99.9);"Bacteroidetes"(98.9);"Bacteroidia"(64.8);"Bacteroidales"(64.8);"Porphyromonadaceae"(58.3);Barnesiella(33.2);
712 Bacteria(100);Firmicutes(100);Clostridia(100);Clostridiales(100);Lachnospiraceae(99.7);Coprococcus(50.1);
713 Bacteria(90);Firmicutes(29.9);Clostridia(25);Clostridiales(22.5);Clostridiales_Incertae_Sedis_XI(5.9);Parvimonas(4.8);
714 Bacteria(86.8);"Bacteroidetes"(23.4);Flavobacteria(9.5);"Flavobacteriales"(9.5);Cryomorphaceae(4.6);Crocinitomix(2.2);
715 Eukaryota(16.7);Streptophyta(11.4);Liliopsida(11.4);Poales(11.4);Poaceae(11.4);Zea(11.4);
716 Eukaryota(13.2);Streptophyta(9.9);Liliopsida(9.9);Poales(9.9);Poaceae(9.9);Zea(9.9);
717 Bacteria(100);Firmicutes(95.4);Clostridia(95.3);Clostridiales(95.3);Eubacteriaceae(28.6);Eubacterium(28.6);
718 Bacteria(100);Firmicutes(96.8);Clostridia(96.3);Clostridiales(96.3);Ruminococcaceae(95.9);Anaerotruncus(66.6);
719 Bacteria(100);Firmicutes(99.9);Clostridia(99.2);Clostridiales(99.2);Clostridiales_Incertae_Sedis_XIII(79.5);Anaerovorax(78.9);
720 Bacteria(100);Firmicutes(95.7);Clostridia(94.7);Clostridiales(94.7);Ruminococcaceae(91.3);Flavonifractor(45.2);
721 Bacteria(88.2);"Deferribacteres"(6.2);Deferribacteres(6.2);Deferribacterales(6.2);Deferribacteraceae(6.2);Mucispirillum(5.9);
722 Bacteria(88.4);Firmicutes(35.5);Clostridia(27.7);Clostridiales(24.8);Clostridiales_Incertae_Sedis_XI(8.3);Parvimonas(6.3);
723 Bacteria(100);Firmicutes(58.2);Clostridia(58.2);Clostridiales(58.2);Ruminococcaceae(58);Oscillibacter(40.8);
724 Bacteria(100);Firmicutes(100);Clostridia(100);Clostridiales(100);Peptostreptococcaceae(100);Clostridium_XI(100);
725 Archaea(18.1);"Euryarchaeota"(14.6);Halobacteria(8.6);Halobacteriales(8.6);Halobacteriaceae(8.6);Halonotius(2.5);
726 Bacteria(100);Firmicutes(100);Clostridia(100);Clostridiales(100);Ruminococcaceae(83.5);Pseudoflavonifractor(62.4);
727 Archaea(24.5);"Euryarchaeota"(20.9);"Methanomicrobia"(16);Methanosarcinales(9.9);Methanosarcinaceae(9.8);Methanohalobium(6.8);
728 Bacteria(100);"Actinobacteria"(95.9);Actinobacteria(95.9);Coriobacteriales(95.9);Coriobacteriaceae(95.9);Enterorhabdus(89.3);
729 Eukaryota(24.9);Chordata(21);Mammalia(21);Rodentia(15.5);Muridae(15.5);Mus(15.5);
730 Eukaryota(27.1);Chordata(26.4);Mammalia(26.4);Rodentia(20);Muridae(20);Mus(20);
731 Eukaryota(19.2);Chordata(16.9);Mammalia(16.9);Rodentia(13);Muridae(13);Mus(13);
732 Bacteria(100);"Bacteroidetes"(10.8);"Bacteroidia"(5.1);"Bacteroidales"(5.1);Bacteroidaceae(5.1);Acetomicrobium(5.1);
733 Archaea(13.7);"Euryarchaeota"(11.5);Halobacteria(10.5);Halobacteriales(10.5);Halobacteriaceae(10.5);Halobaculum(5.6);
734 Bacteria(80.3);Cyanobacteria_Chloroplast(10.8);Chloroplast(10.8);Chloroplast_order_incertae_sedis(10.8);Chloroplast(10.8);Bangiophyceae(10.1);
735 Eukaryota(16.2);Arthropoda(6.8);Insecta(6.8);Coleoptera(6.8);Tenebrionidae(6.8);Tribolium(6.8);
736 Bacteria(100);Firmicutes(99.4);Erysipelotrichia(61.1);Erysipelotrichales(61.1);Erysipelotrichaceae(61.1);Clostridium_XVIII(58.6);
737 Bacteria(100);Firmicutes(92.4);Erysipelotrichia(65.8);Erysipelotrichales(65.8);Erysipelotrichaceae(65.8);Turicibacter(65.7);
738 Bacteria(100);Firmicutes(100);Erysipelotrichia(100);Erysipelotrichales(100);Erysipelotrichaceae(100);Coprobacillus(85.6);
739 Bacteria(100);Firmicutes(100);Clostridia(100);Clostridiales(100);Ruminococcaceae(55);Flavonifractor(21.4);
740 Bacteria(81.9);"Chloroflexi"(7.5);Anaerolineae(5.9);Anaerolineales(5.9);Anaerolineaceae(5.9);Bellilinea(4.3);
741 Bacteria(100);"Bacteroidetes"(100);"Bacteroidia"(100);"Bacteroidales"(100);"Rikenellaceae"(100);Alistipes(100);
742 Eukaryota(13.3);Chordata(10.6);Mammalia(10.6);Rodentia(9.1);Muridae(9.1);Mus(9.1);
743 Bacteria(100);Firmicutes(99.7);Clostridia(99.7);Clostridiales(99.7);Lachnospiraceae(97.7);Marvinbryantia(26.5);
744 Bacteria(75.1);"Proteobacteria"(37.8);Gammaproteobacteria(25.9);Chromatiales(10.8);Halothiobacillaceae(9.9);Thioalkalibacter(9.8);
745 Bacteria(100);Firmicutes(99.8);Clostridia(99.7);Clostridiales(99.7);Clostridiaceae_1(18.5);Anaerosporobacter(18.4);
746 Archaea(19.2);"Euryarchaeota"(16.8);"Methanomicrobia"(11.6);Methanosarcinales(7.1);Methanosarcinaceae(6.6);Methanohalobium(4.5);
747 Bacteria(83.4);"Bacteroidetes"(24.3);"Sphingobacteria"(14.1);"Sphingobacteriales"(14.1);Cytophagaceae(11.4);Litoribacter(4.5);
748 Bacteria(100);Firmicutes(85.1);Clostridia(85);Clostridiales(85);Lachnospiraceae(79.4);Johnsonella(38.5);
749 Eukaryota(8.1);Streptophyta(7.2);Liliopsida(7.2);Poales(7.2);Poaceae(7.2);Zea(7.2);
750 Eukaryota(14.8);Chordata(11.1);Mammalia(11.1);Rodentia(11.1);Muridae(11.1);Mus(11.1);
751 Bacteria(100);Firmicutes(100);Clostridia(100);Clostridiales(100);Lachnospiraceae(99.9);Clostridium_XlVb(49.6);
752 Bacteria(100);"Bacteroidetes"(100);"Bacteroidia"(98.4);"Bacteroidales"(98.4);"Porphyromonadaceae"(81.7);Barnesiella(71);
753 Bacteria(100);Firmicutes(100);Clostridia(100);Clostridiales(100);Lachnospiraceae(99.3);Lachnospiracea_incertae_sedis(50);
754 Bacteria(100);Firmicutes(100);Clostridia(69);Clostridiales(69);Lachnospiraceae(67.6);Pseudobutyrivibrio(53.9);
755 Archaea(16.2);"Euryarchaeota"(13.8);"Methanomicrobia"(8.7);Methanosarcinales(7.9);Methanosarcinaceae(7.9);Methanimicrococcus(7.7);
756 Bacteria(100);Firmicutes(100);Clostridia(100);Clostridiales(100);Lachnospiraceae(93.7);Lachnospiracea_incertae_sedis(36.4);
757 Bacteria(100);"Bacteroidetes"(100);"Bacteroidia"(80.3);"Bacteroidales"(80.3);"Porphyromonadaceae"(64.1);Tannerella(14.8);
758 Eukaryota(10.7);Arthropoda(5.7);Insecta(5.7);Coleoptera(5.7);Tenebrionidae(5.7);Tribolium(5.7);
759 Bacteria(100);Firmicutes(95);Clostridia(95);Clostridiales(95);Ruminococcaceae(94.8);Clostridium_IV(40.8);
760 Bacteria(86.2);"Bacteroidetes"(28.4);"Bacteroidia"(10.1);"Bacteroidales"(10.1);"Porphyromonadaceae"(5.5);Petrimonas(3.7);
761 Bacteria(100);Firmicutes(100);Clostridia(100);Clostridiales(100);Ruminococcaceae(100);Flavonifractor(70.6);
762 Bacteria(100);Firmicutes(99.3);Clostridia(99.3);Clostridiales(99.3);Ruminococcaceae(96.4);Clostridium_IV(38);
763 Bacteria(100);Firmicutes(99.5);Clostridia(99.5);Clostridiales(99.5);Lachnospiraceae(99.3);Shuttleworthia(27.4);
764 Bacteria(100);Firmicutes(100);Clostridia(100);Clostridiales(100);Lachnospiraceae(99.6);Marvinbryantia(36);
765 Bacteria(100);"Bacteroidetes"(97.2);"Bacteroidia"(87);"Bacteroidales"(87);"Porphyromonadaceae"(73.3);Tannerella(35.8);
766 Bacteria(87.8);"Bacteroidetes"(30.2);"Bacteroidia"(9.6);"Bacteroidales"(9.6);"Porphyromonadaceae"(5.9);Petrimonas(4.4);
767 Bacteria(100);"Bacteroidetes"(51.5);"Bacteroidia"(40.5);"Bacteroidales"(40.5);"Porphyromonadaceae"(37.7);Tannerella(27.9);
768 Bacteria(100);"Bacteroidetes"(92);"Bacteroidia"(75.5);"Bacteroidales"(75.5);"Porphyromonadaceae"(69.9);Petrimonas(25.4);
769 Bacteria(100);Firmicutes(99.8);Clostridia(99.8);Clostridiales(99.8);Lachnospiraceae(97.2);Coprococcus(41.5);
770 Eukaryota(14.7);Arthropoda(8.6);Insecta(8.6);Coleoptera(8.6);Tenebrionidae(8.6);Tribolium(8.6);
771 Eukaryota(9.4);Arthropoda(6.9);Insecta(6.9);Coleoptera(6.9);Tenebrionidae(6.9);Tribolium(6.9);

772 Bacteria(100);Firmicutes(99.9);Clostridia(99.9);Clostridiales(99.9);Ruminococcaceae(86.6);Oscillibacter(77.3);
773 Bacteria(100);Firmicutes(99.8);Clostridia(99.8);Clostridiales(99.8);Lachnospiraceae(96.3);Oribacterium(15.9);
774 Bacteria(88.4);"Bacteroidetes"(32.5);Flavobacteria(12.8);"Flavobacteriales"(12.8);Flavobacteriaceae(11.3);Soonwooa(6.7);
775 Bacteria(100);Firmicutes(100);Clostridia(100);Clostridiales(100);Ruminococcaceae(91.4);Oscillibacter(64.6);
776 Bacteria(91.2);"Proteobacteria"(34.6);Alphaproteobacteria(5.4);Rickettsiales(4.1);Anaplasmataceae(4);Wolbachia(4);
777 Eukaryota(12.5);Arthropoda(8.7);Insecta(8.7);Coleoptera(8.7);Tenebrionidae(8.7);Tribolium(8.7);
778 Eukaryota(12.2);Chordata(8.5);Mammalia(8.5);Rodentia(8.5);Muridae(8.5);Mus(8.5);
779 Bacteria(84);"Proteobacteria"(34.1);Alphaproteobacteria(6.3);Rhodospirillales(2.8);Rhodospirillaceae(2.8);Tistrella(2.6);
780 Bacteria(100);Firmicutes(100);Clostridia(100);Clostridiales(100);Lachnospiraceae(99.8);Coprococcus(20.2);
781 Bacteria(100);Firmicutes(84.5);Clostridia(84.2);Clostridiales(84.2);Lachnospiraceae(76.6);Johnsonella(24.4);
782 Bacteria(100);Firmicutes(95.5);Clostridia(95.4);Clostridiales(95.4);Ruminococcaceae(92.2);Anaerotruncus(67.3);
783 Bacteria(100);"Bacteroidetes"(51.1);"Bacteroidia"(46.2);"Bacteroidales"(46.2);"Porphyromonadaceae"(44.8);Barnesiella(40.5);
784 Bacteria(90.2);Firmicutes(30.8);Clostridia(25.6);Clostridiales(23.4);Clostridiales_Incertae_Sedis_XI(6.1);Parvimonas(4.9);
785 Bacteria(100);Firmicutes(97.2);Erysipelotrichia(97.2);Erysipelotrichales(97.2);Erysipelotrichaceae(97.2);Coprobacillus(79.1);
786 Bacteria(100);Firmicutes(85.9);Clostridia(85.9);Clostridiales(85.9);Lachnospiraceae(80.9);Oribacterium(11.3);
787 Bacteria(100);Firmicutes(99.9);Clostridia(99.9);Clostridiales(99.9);Ruminococcaceae(59.8);Flavonifractor(30.1);
788 Bacteria(100);Firmicutes(76.1);Negativicutes(13.1);Selenomonadales(13.1);Veillonellaceae(13.1);Propionispira(2.8);
789 Bacteria(89.8);"Bacteroidetes"(33.8);"Bacteroidia"(11.1);"Bacteroidales"(11.1);"Porphyromonadaceae"(6.2);Petrimonas(3.1);
790 Archaea(26.1);"Euryarchaeota"(23.1);"Methanomicrobia"(7.7);Methanosarcinales(6.6);Methanosarcinaceae(6.6);Methanimicrococcus(6.5);
791 Bacteria(100);"Bacteroidetes"(99.9);"Bacteroidia"(98.6);"Bacteroidales"(98.6);"Porphyromonadaceae"(95.2);Tannerella(38.1);
792 Bacteria(100);Firmicutes(94.5);Clostridia(94.4);Clostridiales(94.4);Ruminococcaceae(92);Acetanaerobacterium(86.9);
793 Bacteria(100);Firmicutes(98);Clostridia(96.5);Clostridiales(96.5);Ruminococcaceae(56.1);Acetanaerobacterium(20.2);
794 Bacteria(100);Firmicutes(99.6);Clostridia(95.5);Clostridiales(95);Ruminococcaceae(43.8);Saccharofermentans(42.8);
795 Bacteria(100);"Bacteroidetes"(75.8);"Bacteroidia"(66.9);"Bacteroidales"(66.9);"Porphyromonadaceae"(43);Barnesiella(30.5);
796 Bacteria(100);"Bacteroidetes"(91);"Bacteroidia"(80.6);"Bacteroidales"(80.6);"Porphyromonadaceae"(66.9);Tannerella(43);
797 Bacteria(89.5);"Proteobacteria"(34.2);Alphaproteobacteria(14.2);Rhizobiales(5.9);Bradyrhizobiaceae(4.1);Agromonas(4.1);
798 Bacteria(100);Firmicutes(67.1);Clostridia(67.1);Clostridiales(67.1);Lachnospiraceae(62.3);Johnsonella(44.8);
799 Bacteria(85.7);"Bacteroidetes"(25.2);"Bacteroidia"(6.2);"Bacteroidales"(6.2);"Porphyromonadaceae"(3.4);Petrimonas(1.4);
800 Bacteria(100);Firmicutes(56.9);Clostridia(32.2);Clostridiales(30);Ruminococcaceae(19);Saccharofermentans(16.8);
801 Bacteria(100);Firmicutes(100);Clostridia(100);Clostridiales(100);Lachnospiraceae(99.9);Lactonifactor(14.6);
802 Bacteria(100);Firmicutes(98.9);Clostridia(98.9);Clostridiales(98.8);Lachnospiraceae(97.6);Marvinbryantia(32.7);
803 Bacteria(100);Firmicutes(99.7);Clostridia(99.4);Clostridiales(99.4);Clostridiaceae_1(76);Sarcina(55.9);
804 Bacteria(82.2);"Proteobacteria"(34.4);Gammaproteobacteria(19.8);Chromatiales(10.6);Halothiobacillaceae(9.9);Thioalkalibacter(9.9);
805 Bacteria(87.2);"Proteobacteria"(29.8);Deltaproteobacteria(10.1);Syntrophobacterales(4.9);Syntrophaceae(3.3);Smithella(3.3);
806 Bacteria(100);Firmicutes(64.6);Clostridia(64.6);Clostridiales(64.6);Lachnospiraceae(62.5);Marvinbryantia(16.8);
807 Bacteria(100);Firmicutes(99.9);Clostridia(99.9);Clostridiales(99.9);Ruminococcaceae(99.9);Oscillibacter(99.3);
808 Bacteria(100);"Bacteroidetes"(100);"Bacteroidia"(99.6);"Bacteroidales"(99.6);"Porphyromonadaceae"(93.2);Tannerella(71);
809 Bacteria(100);Firmicutes(100);Clostridia(100);Clostridiales(100);Lachnospiraceae(99.6);Moryella(38.5);
810 Bacteria(100);Firmicutes(100);Clostridia(100);Clostridiales(100);Ruminococcaceae(100);Clostridium_IV(42.9);
811 Eukaryota(12.9);Arthropoda(8.5);Insecta(8.5);Coleoptera(8.5);Tenebrionidae(8.5);Tribolium(8.5);
812 Bacteria(100);"Bacteroidetes"(77.3);"Bacteroidia"(41.9);"Bacteroidales"(41.9);"Porphyromonadaceae"(39.7);Tannerella(22.8);
813 Bacteria(99.9);Firmicutes(88.9);Clostridia(88.9);Clostridiales(88.9);Ruminococcaceae(78);Anaerotruncus(45.2);
814 Eukaryota(10.6);Arthropoda(6);Insecta(6);Coleoptera(6);Tenebrionidae(6);Tribolium(6);
815 Eukaryota(15.5);Arthropoda(9.6);Insecta(9.6);Coleoptera(9.6);Tenebrionidae(9.6);Tribolium(9.6);
816 Bacteria(100);Firmicutes(100);Clostridia(100);Clostridiales(100);Ruminococcaceae(59);Oscillibacter(51.5);
817 Bacteria(76.6);"Proteobacteria"(21.6);Deltaproteobacteria(10.7);Myxococcales(4.8);Nannocystaceae(4.7);Enhygromyxa(4.7);
818 Bacteria(100);Firmicutes(97.8);Clostridia(80.7);Clostridiales(80.7);Lachnospiraceae(70.7);Catonella(12);
819 Bacteria(99.6);"Bacteroidetes"(81.5);Flavobacteria(22.8);"Flavobacteriales"(22.8);Cryomorphaceae(21.6);Fluviicola(20.9);
820 Bacteria(100);Firmicutes(100);Clostridia(100);Clostridiales(100);Lachnospiraceae(98.9);Lachnospiracea_incertae_sedis(49.1);
821 Bacteria(100);Firmicutes(98.8);Clostridia(98.8);Clostridiales(98.8);Lachnospiraceae(98.1);Lactonifactor(39.7);
822 Bacteria(100);Firmicutes(82.8);Clostridia(80.6);Clostridiales(80.4);Ruminococcaceae(64);Flavonifractor(9.9);
823 Bacteria(86.1);"Thermodesulfobacteria"(5);Thermodesulfobacteria(5);Thermodesulfobacteriales(5);Thermodesulfobacteriaceae(5);Caldimicrobium(5);
824 Eukaryota(9.5);Arthropoda(6);Insecta(6);Coleoptera(6);Tenebrionidae(6);Tribolium(6);
825 Archaea(21.1);"Euryarchaeota"(18.7);Halobacteria(11.2);Halobacteriales(11.2);Halobacteriaceae(11.2);Halonotius(5.2);
826 Bacteria(100);"Bacteroidetes"(99.8);"Bacteroidia"(93.8);"Bacteroidales"(93.8);"Porphyromonadaceae"(65.2);Barnesiella(50.6);
827 Bacteria(99.9);Firmicutes(88.2);Clostridia(88);Clostridiales(88);Lachnospiraceae(83.4);Marvinbryantia(21.3);
828 Bacteria(100);Firmicutes(100);Clostridia(100);Clostridiales(100);Ruminococcaceae(100);Oscillibacter(94.2);
829 Eukaryota(9.7);Streptophyta(8.6);Liliopsida(8.6);Poales(8.6);Poaceae(8.6);Zea(8.6);
830 Bacteria(90);"Bacteroidetes"(24.3);"Bacteroidia"(8);"Bacteroidales"(8);"Porphyromonadaceae"(3.3);Petrimonas(1.7);
831 Bacteria(80.3);"Proteobacteria"(38.3);Gammaproteobacteria(24.7);Chromatiales(10.2);Halothiobacillaceae(9.4);Thioalkalibacter(9.3);
832 Bacteria(100);Firmicutes(96.8);Clostridia(87.3);Clostridiales(79.5);Ruminococcaceae(71.4);Papillibacter(22.1);
833 Bacteria(100);"Bacteroidetes"(99.9);"Bacteroidia"(55.1);"Bacteroidales"(55.1);"Rikenellaceae"(24.5);Rikenella(24.5);
834 Bacteria(100);Firmicutes(96.6);Clostridia(93.7);Clostridiales(93.3);Lachnospiraceae(71.6);Robinsoniella(21.4);
835 Bacteria(89.5);"Elusimicrobia"(2.9);Elusimicrobia(2.9);Elusimicrobiales(2.9);Elusimicrobiaceae(2.9);Elusimicrobium(2.9);
836 Bacteria(100);Firmicutes(100);Clostridia(100);Clostridiales(100);Ruminococcaceae(100);Oscillibacter(90.8);
837 Bacteria(89.7);"Proteobacteria"(40.1);Alphaproteobacteria(18.4);Rhizobiales(5.9);Bradyrhizobiaceae(3.8);Agromonas(3.7);
838 Eukaryota(12.8);Chordata(7.1);Mammalia(7.1);Primates(1.8);Hominidae(1.8);Homo(1.8);
839 Bacteria(100);Firmicutes(100);Clostridia(100);Clostridiales(100);Lachnospiraceae(99.4);Syntrophococcus(30.2);
840 Bacteria(100);"Bacteroidetes"(100);"Bacteroidia"(99.7);"Bacteroidales"(99.7);"Porphyromonadaceae"(98.2);Porphyromonas(30.4);
841 Bacteria(79.3);Firmicutes(28.6);Clostridia(21.7);Clostridiales(17.3);Incertae_Sedis_XI(5.9);Murdochiella(5.9);
842 Bacteria(100);Firmicutes(98.3);Clostridia(98);Clostridiales(98);Ruminococcaceae(91.1);Pseudoflavonifractor(53.9);
843 Bacteria(100);Firmicutes(100);Clostridia(100);Clostridiales(100);Ruminococcaceae(86.2);Clostridium_IV(68.5);
844 Eukaryota(13.2);Chordata(11.4);Mammalia(11.4);Rodentia(11.4);Muridae(11.4);Mus(11.4);
845 Bacteria(100);Firmicutes(93.2);Clostridia(89.2);Clostridiales(86.9);Ruminococcaceae(52.1);Saccharofermentans(31.7);
846 Bacteria(100);Firmicutes(56.9);Clostridia(56.1);Clostridiales(56.1);Ruminococcaceae(48.7);Flavonifractor(34.8);
847 Bacteria(100);Firmicutes(100);Clostridia(100);Clostridiales(100);Lachnospiraceae(98.3);Lachnospiracea_incertae_sedis(42.9);
848 Bacteria(100);Firmicutes(100);Clostridia(100);Clostridiales(100);Ruminococcaceae(100);Oscillibacter(85.1);
849 Eukaryota(14.1);Arthropoda(7);Insecta(7);Coleoptera(7);Tenebrionidae(7);Tribolium(7);
850 Eukaryota(20);Streptophyta(8);Liliopsida(8);Poales(8);Poaceae(8);Zea(8);
851 Bacteria(100);"Bacteroidetes"(92.8);"Bacteroidia"(91.5);"Bacteroidales"(91.5);"Porphyromonadaceae"(70.1);Barnesiella(39.2);
852 Bacteria(99.3);"Bacteroidetes"(93);"Bacteroidia"(69);"Bacteroidales"(69);"Porphyromonadaceae"(59.1);Tannerella(30.2);
853 Archaea(20.3);"Euryarchaeota"(17.2);Halobacteria(9.2);Halobacteriales(9.2);Halobacteriaceae(9.2);Halonotius(3.9);
854 Bacteria(100);Firmicutes(99.6);Clostridia(99.6);Clostridiales(99.6);Lachnospiraceae(93.7);Lactonifactor(27.4);
855 Bacteria(100);Firmicutes(56);Clostridia(53.7);Clostridiales(53.7);Clostridiaceae_1(46.2);Anaerobacter(44.6);
856 Bacteria(85.2);"Actinobacteria"(9.6);Actinobacteria(9.6);Actinomycetales(9);Micromonosporaceae(6.6);Catelliglobosispora(6.3);
857 Bacteria(86.9);"Bacteroidetes"(28.5);"Bacteroidia"(10.1);"Bacteroidales"(10.1);"Porphyromonadaceae"(5.3);Petrimonas(2.8);

858 Bacteria(100);"Actinobacteria"(100);Actinobacteria(100);Coriobacteriales(100);Coriobacteriaceae(100);Enterorhabdus(100);
859 Eukaryota(5);Chordata(4.1);Mammalia(4.1);Rodentia(4.1);Muridae(4.1);Mus(4.1);
860 Bacteria(87.6);"Proteobacteria"(28.9);Deltaproteobacteria(9.2);Desulfobacterales(5.3);Desulfobacteraceae(2.6);Desulfatiferula(2.2);
861 Bacteria(100);Firmicutes(100);Bacilli(100);Lactobacillales(100);Lactobacillaceae(100);Lactobacillus(100);
862 Eukaryota(12.8);Chordata(9.7);Mammalia(9.7);Rodentia(9.7);Muridae(9.7);Mus(9.7);
863 Bacteria(100);Firmicutes(100);Clostridia(100);Clostridiales(99.8);Ruminococcaceae(97.1);Oscillibacter(82.3);
864 Eukaryota(25.9);Chordata(24.4);Mammalia(24.4);Rodentia(18.8);Muridae(18.8);Mus(18.8);
865 Bacteria(100);Firmicutes(100);Clostridia(86.3);Clostridiales(86.3);Ruminococcaceae(86);Flavonifractor(57.2);
866 Archaea(18.7);"Euryarchaeota"(15.7);"Methanomicrobia"(8.1);Methanosarcinales(6.5);Methanosarcinaceae(6.5);Methanimicrococcus(6.5);
867 Bacteria(100);Firmicutes(99.3);Clostridia(97.1);Clostridiales(97.1);Ruminococcaceae(66.7);Oscillibacter(23.4);
868 Archaea(18.5);"Euryarchaeota"(15.1);"Methanomicrobia"(7.2);Methanosarcinales(4.3);Methanosarcinaceae(4.2);Methanohalobium(2.2);
869 Eukaryota(18.8);Chordata(14);Mammalia(14);Rodentia(14);Muridae(14);Mus(14);
870 Archaea(10.5);"Euryarchaeota"(9.7);Thermoplasmata(6.7);Thermoplasmatales(6.7);Ferroplasmaceae(6.5);Ferroplasma(6.5);
871 Eukaryota(16.9);Arthropoda(10.8);Insecta(10.8);Coleoptera(10.8);Tenebrionidae(10.8);Tribolium(10.8);
872 Eukaryota(12.5);Arthropoda(8);Insecta(8);Coleoptera(8);Tenebrionidae(8);Tribolium(8);
873 Archaea(18.3);"Euryarchaeota"(14.8);Halobacteria(10.1);Halobacteriales(10.1);Halobacteriaceae(10.1);Halonotius(3.8);
874 Bacteria(100);"Bacteroidetes"(99.9);"Bacteroidia"(94.2);"Bacteroidales"(94.2);"Porphyromonadaceae"(93.6);Barnesiella(83.2);
875 Bacteria(100);Firmicutes(100);Clostridia(100);Clostridiales(100);Lachnospiraceae(96.9);Clostridium_XlVa(82.9);
876 Eukaryota(11.9);Streptophyta(10.8);Liliopsida(10.8);Poales(10.8);Poaceae(10.8);Zea(10.8);
877 Eukaryota(24.4);Chordata(23.6);Mammalia(23.6);Rodentia(18.2);Muridae(18.2);Mus(18.2);
878 Bacteria(83.2);"Thermodesulfobacteria"(4.4);Thermodesulfobacteria(4.4);Thermodesulfobacteriales(4.4);Thermodesulfobacteriaceae(4.4);Caldimicrobium(4.4);
879 Bacteria(89);"Proteobacteria"(36.7);Alphaproteobacteria(5.2);Rickettsiales(3.3);Anaplasmataceae(3.3);Wolbachia(3.3);
880 Eukaryota(17.1);Chordata(10.9);Mammalia(10.9);Rodentia(10.9);Muridae(10.9);Mus(10.9);
881 Bacteria(100);Firmicutes(100);Negativicutes(100);Selenomonadales(100);Acidaminococcaceae(96.8);Phascolarctobacterium(84.2);
882 Eukaryota(10.8);Arthropoda(6.1);Insecta(6.1);Coleoptera(6.1);Tenebrionidae(6.1);Tribolium(6.1);
883 Bacteria(86.4);Firmicutes(32.4);Clostridia(27.7);Clostridiales(24.9);Clostridiales_Incertae_Sedis_XI(10.3);Parvimonas(8.3);
884 Bacteria(100);Firmicutes(99.4);Clostridia(99.4);Clostridiales(99.4);Lachnospiraceae(89.7);Marvinbryantia(28.6);
885 Bacteria(89);"Proteobacteria"(34.8);Alphaproteobacteria(15);Rhizobiales(6.2);Bradyrhizobiaceae(4.2);Agromonas(4.1);
886 Bacteria(90);"Proteobacteria"(33.6);Epsilonproteobacteria(6.4);Campylobacterales(6.2);"Hydrogenimonaceae"(5.6);Hydrogenimonas(5.6);
887 Bacteria(100);Firmicutes(100);Clostridia(100);Clostridiales(100);Peptostreptococcaceae(100);Clostridium_XI(92);
