# Supplementary material for: Diet and exercise orthogonally alter the gut microbiome and reveal independent associations with anxiety and cognition
Source: Mol Neurodegener. 2014 Sep 13;9:36. doi: 10.1186/1750-1326-9-36 (PMC4168696; doi:10.1186/1750-1326-9-36)
Supplement: Additional file 3: Table S1 — Significant effects of diet and exercise at different levels of taxonomy. *P<0.05, **P<0.01, ***P<0.001, ****P<0.0001. [file 1750-1326-9-36-S3.pdf]

Supplemental Table S1. Significant effects of diet and exercise at different levels of taxonomy.

	Main effects 2-way ANOVA			post-hoc testing			
	High fat diet	exercise	interaction	ND vs HFD	ND+ex vs HFD+ex	ND vs ND+ex	HFD vs HFD+ex
**Phylum**							
Bacteroidetes	↓ ****	↓ **		****	****	*	*
Firmicutes	↑ ****	↑ **		****	****	*	*
Tenericutes	↓ **		*	**		*	
Proteobacteria	↑ *	↑ *					
Actinobacteria		↑ *					
**Class**							
Bacteroidia	↓ ****	↓ ***		****	****	*	*
Clostridia	↑ ****	↑ **		****	****	*	*
Erysipelotrichia	↓ ****			**	***		
Mollicutes	↓ **		*	**		*	
Bacilli	↑ **						
Gammaproteobacteria	↑ *						
Negativicutes	↑ *						
Betaproteobacteria		↑ **					*
Actinobacteria		↑ *					
Sphingobacteria		↑ *			*		*
Flavobacteria		↑ *					*
Alphaproteobacteria		↑ *					*
**Order**							
Bacteroidales	↓ ****	↓ ***		****	****	*	*
Clostridiales	↑ ****	↑ **		****	****	*	*
Erysipelotrichales	↓ ****			**	***		
Lactobacillales	↑ ***				*		
Anaeroplasmatales	↓ **		*	**		*	
Bacillales	↓ **						
Pseudomonadales	↑ *	↑ *			*		*
Enterobacteriales	↑ *						
Selenomonadales	↑ *						
Burkholderiales		↑ **					*
Actinomycetales		↑ *					
Coriobacteriales		↑ *				*	
Sphingobacteriales		↑ *			*		*
Flavobacteriales		↑ *					*
Caulobacterales		↑ *					*
Rhizobiales		↑ *					*
