# Supplementary material for: Diet and exercise orthogonally alter the gut microbiome and reveal independent associations with anxiety and cognition
Source: Mol Neurodegener. 2014 Sep 13;9:36. doi: 10.1186/1750-1326-9-36 (PMC4168696; doi:10.1186/1750-1326-9-36)
Supplement: Additional file 4: Figure S2 — Anxiety levels were associated with several bacteria at the level of phyla, class, order, and family. Color scheme of data points follows the format as in Figure 1 (ND = blue, ND + exercise = green, HFD = red, and HFD + exercise in orange). Linear regression analysis for individual mice was performed with R2 values indicating goodness of fit and uncorrected p values for slope calculated by F test. [file 1750-1326-9-36-S4.pdf]

# Supplemental Figure S2

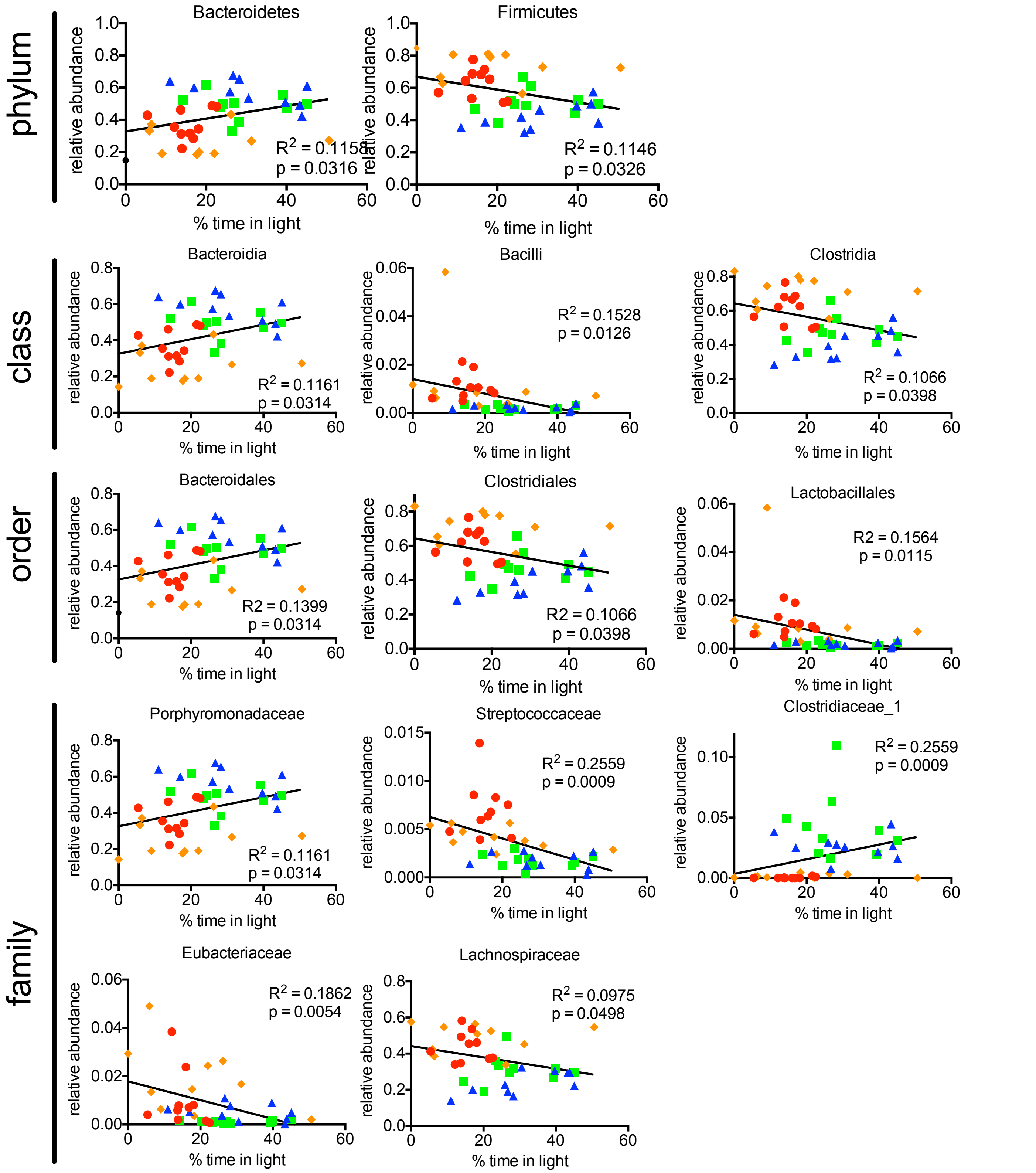


Figure S2. Anxiety levels were associated with several bacteria at the level of phyla, class, order, and family. Color scheme of data points follows the format as in Figure 1 (ND = blue, ND + exercise = green, HFD = red, and HFD + exercise in orange). Linear regression analysis for individual mice was performed with $R^2$ values indicating goodness of fit and uncorrected p values for slope calculated by F test.
