# Supplementary material for: Diet and exercise orthogonally alter the gut microbiome and reveal independent associations with anxiety and cognition
Source: Mol Neurodegener. 2014 Sep 13;9:36. doi: 10.1186/1750-1326-9-36 (PMC4168696; doi:10.1186/1750-1326-9-36)
Supplement: Additional file 5: Table S3 — Associations of bacterial abundances with body weight. *P<0.05, **P<0.01, ***P<0.001, ****P<0.0001. [file 1750-1326-9-36-S5.pdf]

Supplemental Table S3. Associations of bacterial abundances with body weight.

taxa	Slope	R square	p value
Bacteroidetes	-0.002756 ± 0.0006099	0.3495	0.0001
Firmicutes	0.002759 ± 0.0006103	0.3498	0.0001
Bacteroidia	-0.002776 ± 0.0006155	0.3486	0.0001
Bacilli	0.0001799 ± 4.064e-005	0.3403	0.0001
Clostridia	0.002896 ± 0.0006277	0.3591	0.0001
Erysipelotrichia	-0.0003175 ± 7.042e-005	0.3485	0.0001
Bacteroidales	-0.002776 ± 0.0006155	0.3486	0.0001
Lactobacillales	0.0001825 ± 4.060e-005	0.3471	0.0001
Clostridiales	0.002896 ± 0.0006277	0.3591	0.0001
Erysipelotrichales	-0.0003175 ± 7.042e-005	0.3485	0.0001
Streptococcaceae	6.102e-005 ± 1.012e-005	0.489	0.0001
Clostridiaceae_1	-0.0004780 ± 9.020e-005	0.4249	0.0001
Lachnospiraceae	0.002492 ± 0.0005078	0.388	0.0001
Porphyromonadaceae	-0.002776 ± 0.0006156	0.3486	0.0001
Erysipelotrichaceae	-0.0003175 ± 7.042e-005	0.3485	0.0001
Ruminococcaceae	0.0005949 ± 0.0001923	0.2012	0.0037
Lactobacillaceae	0.0001214 ± 3.995e-005	0.1955	0.0043
Clostridiales_Incertae_Sedis_XIII	8.926e-007 ± 3.416e-007	0.1558	0.0129
Microbacteriaceae	7.868e-008 ± 3.026e-008	0.1544	0.0133
Bacillales	-2.547e-006 ± 1.005e-006	0.1446	0.0155
Gammaproteobacteria	1.005e-005 ± 3.993e-006	0.143	0.0161
Proteobacteria	1.269e-005 ± 5.423e-006	0.1259	0.0246
Staphylococcaceae	-2.073e-006 ± 8.873e-007	0.1256	0.0248
Bacteroidaceae	2.696e-007 ± 1.184e-007	0.123	0.0286
Xanthomonadaceae	1.076e-006 ± 4.842e-007	0.1178	0.0324
Enterobacteriales	2.106e-006 ± 9.845e-007	0.1075	0.0389
Enterobacteriaceae	2.106e-006 ± 9.845e-007	0.1075	0.0389
Rhizobiales	5.527e-007 ± 2.618e-007	0.105	0.0414

Benjamini-Hochberg p value
0.00280
0.00140
0.00093
0.00070
0.00056
0.00047
0.00040
0.00035
0.00031
0.00028
0.00025
0.00023
0.00022
0.00020
0.00019
0.00648
0.00708
0.02007
0.01960
0.02170
0.02147
0.03131
0.03019
0.03337
0.03629
0.04189
0.04034
0.04140
